# Visual explainable artificial intelligence for graph-based visual question answering and scene graph curation

**DOI:** 10.1186/s42492-025-00185-y

**Published:** 2025-04-07

**Authors:** Sebastian Künzel, Tanja Munz-Körner, Pascal Tilli, Noel Schäfer, Sandeep Vidyapu, Ngoc Thang Vu, Daniel Weiskopf

**Affiliations:** 1 VISUS, University of Stuttgart, Stuttgart, 70569 Germany; 2 IMS, University of Stuttgart, Stuttgart, 70569 Germany

**Keywords:** Visual question answering, Explainable artificial intelligence, Visual analytics, Scene graphs

## Abstract

This study presents a novel visualization approach to explainable artificial intelligence for graph-based visual question answering (VQA) systems. The method focuses on identifying false answer predictions by the model and offers users the opportunity to directly correct mistakes in the input space, thus facilitating dataset curation. The decision-making process of the model is demonstrated by highlighting certain internal states of a graph neural network (GNN). The proposed system is built on top of a GraphVQA framework that implements various GNN-based models for VQA trained on the GQA dataset. The authors evaluated their tool through the demonstration of identified use cases, quantitative measures, and a user study conducted with experts from machine learning, visualization, and natural language processing domains. The authors’ findings highlight the prominence of their implemented features in supporting the users with incorrect prediction identification and identifying the underlying issues. Additionally, their approach is easily extendable to similar models aiming at graph-based question answering.

**Supplementary Information:**

The online version contains supplementary material available at 10.1186/s42492-025-00185-y.

## Introduction

Many state-of-the-art systems for computer vision (CV) and natural language processing (NLP) tasks are based on modern deep learning (DL) algorithms. Visual question answering (VQA) requires techniques from both areas owing to its inherent design at the intersection of NLP and CV. The task involves asking the model to predict an answer for a given question-image pair from a pre-defined set of answers. Consequently, VQA is mostly treated as a supervised classification problem.

A particular case of VQA involves scene graphs as visual input [1] as opposed to images. Recently, scene graphs have become a popular means in VQA tasks [2] and offer a more abstract and structured representation of objects and their relationships to the scene depicted in an image.

The authors build on top of a system designed for graph-based VQA. GraphVQA [3] is a framework that utilizes graph neural networks (GNNs) to process scene graphs to answer questions related to arbitrary concepts that can be found in the visual scene. Consequently, the input consists of a scene graph and a natural language question. The system then predicts a distribution of confidence scores for each possible answer category from a closed set. The proposed approach leverages ground-truth scene graphs contained within the GQA dataset [4], which comprises approximately 22 million questions on 113,000 unique images. The scene graphs highlight key objects (e.g., a bird and a flower) and their associated properties (e.g., color) as well as directed pairwise relations among objects in natural language (e.g., relative position, size, or order). To aid in training and validation, each question is paired with precisely one expected answer.

Many challenges arise when designing, training, and evaluating such VQA systems for deployment in real-world scenarios. This study focuses on the evaluation and analysis of existing graph-based VQA models. The authors investigate how to aid developers to better understand the model and improve its performance via a visual analytics application. As previously demonstrated, such systems suffer from various problems including dataset quality and biases [5] and explainability.

Explainable artificial intelligence (XAI) is concerned with methods to explain the decision-making problem of black box machine learning (ML) methods. Despite the impressive results of artificial neural networks (NNs), many DL-based approaches are considered black boxes [6–8] by users and developers, making it challenging to deploy them in high-stakes domains such as healthcare, medicine, or autonomous driving.

**Fig. 1:**
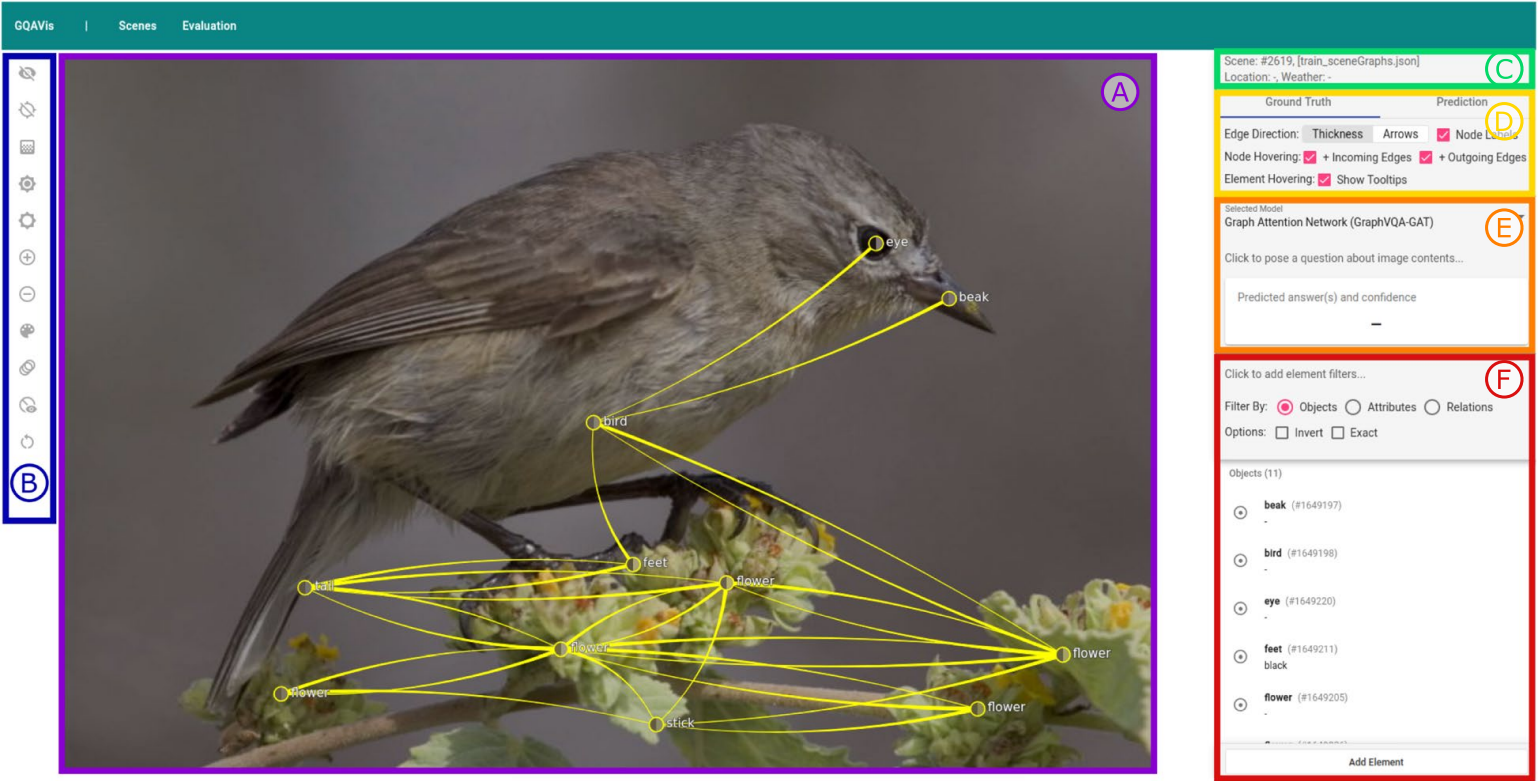
Overview of the proposed visual tool: A ground truth scene is shown in the visual analysis system. The scene graph is drawn on top of image (**A**) with different options for the visibility of the image and scene graph (**B**). Several pieces of information and settings are available in the right panel of the visual analysis system: meta-information about the current scene (**C**), additional settings for the visualization (**D**), an area for asking a question (**E**), a list of all objects and relations of the scene graph with filtering options, and the possibility to add new elements (**F**). Background image: GQA dataset [4] (scene 2619) under Creative Commons CC BY 4.0

To address the aforementioned issue, the authors propose a visual analytics approach that allows users and developers to gain insight into the reasoning process of a scene graph question answering (Scene Graph QA) system based on GNNs. Their tool facilitates dataset curation and model explanation, thus addressing XAI via interactive visualization (Fig. 1). Owing to the multi-modality of VQA, it is crucial to fuse scene data, question data, and answer tokens into one visualization. In the showcased visual analysis tool, the authors integrate the model, scene data, and user input. This allows us to observe information propagation during message passing in GNNs. The authors encourage the user to modify both scene data and input questions to steer the model’s attention.

The tool is designed to generalize to similar models, i.e., it is easily extensible to include new types of models. The visualization requires two main ingredients from the GNN: (1) scores or weights from the aggregation method that defines how the node embeddings are fused to a single graph embedding, and (2) scores or weights for each edge in the scene graph. Because the authors deal with a graph classification problem, the learned node embeddings can be aggregated into a single embedding vector representing the whole graph. They leverage this information to determine the corresponding node size in the visualization. The weights on the edge level are an inherent element of GNNs designed on the message-passing paradigm. Exchanging the GNN can thereby be achieved easily without any drawbacks to the visualization.

The major contributions of this study include:

1. Visual analytics approach to XAI for graph-based VQA systems.
2. Evaluation of the key features of the authors’ implemented tool through different types of evaluation: use cases, quantitative evaluation, user study.

This work is an extension of the authors’ previous paper [9]. The authors extended their earlier system by filtering options in the user interface and included several changes and options for the visual representation of the current scene (i.e. visibility, color, and size of elements). Compared to their previous paper, this paper provides additional technical details about the underlying model, more information about the different components of their system (the scene browser, evaluation browser, and ground truth visualization), an in-depth demonstration of use cases of their approach, a user study assessing the usefulness of their approach, and an updated discussion of directions for future work. Furthermore, they re-wrote large portions of the original paper, replaced images from the visualization system by different examples, and added several new images.

In the supplemental video, the authors demonstrate the interactive capabilities of their tool. The source code of their system is publicly available on DaRUS [10] and GitHub.^1^

### Related work

Visualization and visual analytics [11, 12] are helpful approaches to increase the transparency and interpretability of DL models, facilitate debugging of predictions, and improve model quality through user input. There are several survey papers on visual analytics for ML and DL [13–16]. For instance, Choo and Liu [17] reviewed visual analytics solutions under the paradigm of explainable DL, emphasizing how interactive visualizations promote intuitive understanding of models, including those that visualize the training or inference process. The proposed approach focuses on the latter.

Several visual analytics systems have been designed for different application domains within NLP: ActiVis [18] enables the exploration of NNs with a focus on neuron activation patterns. Strobelt et al. [19] presented LSTMVis, a tool supporting visual analysis of hidden state dynamics and interpretation of feature representation in long short-term memory (LSTM). RNNVis [20] is a visual analytics system for analyzing hidden state changes of recurrent neural networks that provide insight into how the supported models process and store information similarly or dissimilarly for different inputs. NMTVis [21] is a visual analysis solution targeting LSTM- and Transformer-based DL models for machine translation. Erroneous sentences can be selected, analyzed, and modified. The system by Garcia et al. [22] visualizes the hidden states of ML models, specifically LSTM-based NLP ones.

In contrast to the aforementioned papers, the proposed approach is tailored to graph-based VQA. VQA [23] is a rapidly expanding research area, with many recent methods and datasets that enhance performance [24]. However, like other areas of NLP, the interpretability of the models remains a critical issue. Many existing studies use pixel-based visualizations that highlight areas where the model focuses most while answering a question. These approaches are based on different heat maps plotted on top of an image. They can be applied as attention maps of NNs with attention mechanism [25] or Transformers [26] to highlight areas with the highest attention of specific layers. Goyal et al. [27] used an importance map on an image and highlighted important words in the question using guided backpropagation. Class activation mapping, such as Grad-CAM of CNNs can show the importance of each region of the image related to the decision-making process of the model [28]. Others show error maps to highlight areas that might be erroneous [29] or use multiple maps for different VQA systems to generate a final heat map [30]. Furthermore, approaches have been designed to explicitly improve interpretability. The model by Norcliffe-Brown et al. [31] learns a graph structure from the question. Subsequently, bounding boxes as nodes and edges between nodes are visualized on top of an image to indicate the most relevant objects and edges concerning the question, enhancing transparency. Compared to these papers, graph-based VQA does not directly predict based on the image data but on scene graphs [32]. Ghosh et al. [33] generated explanations in natural language from a scene graph and an attention map. However, to the best of the authors’ knowledge, no existing work provides a visual analysis approach for VQA based on scene graphs.

As described in the introduction, this work is an extension of our previous paper [9]. Preliminary work for that earlier paper was done in a master’s thesis [34].

### Technical background

Here, the authors introduce the key aspects of this work. They first introduce VQA [23] before highlighting the differences to graph-based VQA. Thereafter, they go into detail about GNNs in general and explain the aspects that they visualized.

### VQA

**Fig. 2:**
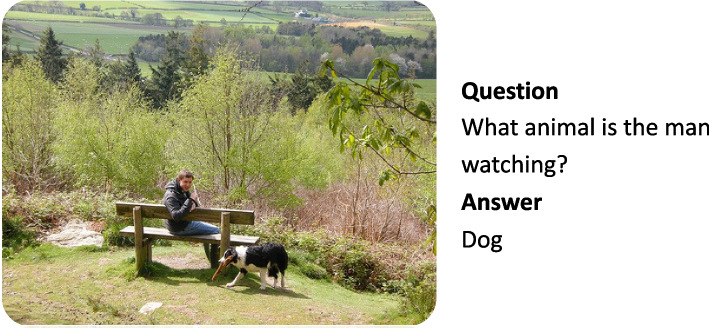
Example image-question pair with the correct answer from the GQA dataset [4] under Creative Commons CC BY 4.0

VQA is a sub-field of NLP and CV. Given pairs of images and a natural language questions, a VQA system automatically generates answers for the questions. An exemplary data sample including image, question, and answer is displayed in Fig. 2.

Typically, VQA systems rely on DL approaches [35]: the image and question are separately processed to extract features, that are then used to generate the final prediction for the answer. Often, attention mechanisms are used to focus on the most important areas of images and the questions. VQA is treated as a classification problem, i.e., the model predicts an answer from a predefined set of answers [36]. The system is optimized using a cross-entropy loss function, which results in learning a probability distribution over possible answer. The answer with the highest probability or confidence score is selected as the final prediction. For training, a large amount of images, questions, and corresponding answers are required.

The proposed approach is based on graph-based VQA [2], a novel approach for enhancing the understanding of the visual content. Here, images are not directly fed into the model. Instead, a scene graph [1] representation of each image is required. Scene graphs are directed node-link diagrams consisting of nodes and edges that describe the content of the image. The nodes represent the objects in an image with their attributes, and the edges describe relationships among neighboring objects. To process such scene graphs, GNNs are leveraged, which are special types of NNs designed to process graphs. In the proposed approach, a Graph attention network (GAT) is employed, where each edge is assigned an attention value that can be used in the analysis to understand the prediction mechanism better. More details about GNNs follow in the next subsection.

### GNN visualization

One of the authors’ main objectives in the visualization approach is to display internal states of GNNs to users. Consequently, they provide background information on the basic concept of GNNs. Specifically, they focus on the information propagation via edge weights and node attention, which can be directly accessed in their visualization to increase the understanding of the decision-making process and support users in identifying issues during the prediction.

#### GNNs

GNNs are an extension of DL-based methods for graph data that pose the challenge of having irregular sizes. A directed graph $\mathcal {G}=(\mathcal {V},\mathcal {E})$ is defined consisting of a set of nodes $\mathcal {V}$ and a set of edges $\mathcal {E}$. Each node $v_i \in \mathcal {V}$ defines a node; $e_{i,j} = (v_i, v_j) \in \mathcal {E}$ defines an edge between nodes $v_i$ and $v_j$. The neighbors of $v_i$ are defined as $\mathcal {N}(v_i) = \{ v_j \in \mathcal {V} | e_{i,j} \in \mathcal {E}\}$. A GNN is defined by the way it aggregates the information of its neighbors $\mathcal {N}(v_i)$ to update a node representation vector $\bf x_i$, as displayed in Eq. 1:

1 $$\begin{aligned} \textbf{x}_i^{\prime }= \texttt{Update}\left( \textbf{x}_i, \texttt{Aggregate}\left(\left\{\textbf{x}_j, \forall j \in \mathcal {N}(v_i)\right\}\right) \right) \end{aligned}$$

Update and Aggregate are arbitrarily differentiable functions, typically NNs [37].

#### Layer-wise edge weight visualization

In their visualization, the authors include the option to provide a scalar for each edge, indicating its importance. Because GNNs aggregate the hidden states of its neighbor nodes, this information can be leveraged to highlight certain edges over others. For example, in a graph convolution network [38] as displayed in Eq. 2, every edge is weighted equally during the updating step:

2 $$\begin{aligned} \textbf{X}^{\prime } = \hat{\textbf{A}} \textbf{X} \varvec{\Theta }\, \end{aligned}$$

where $\hat{\textbf{A}}$ is the adjacency matrix (containing the information on nodes connected via edges), $\textbf{X}$ containing the corresponding node embeddings, and $\varvec{\Theta }$ are the trainable parameters. In contrast, in a GAT [39], every edge can be weighted differently via a learnable attention weight $\alpha _{i,j}$:

3 $$\begin{aligned} \textbf{x}^{\prime }_i = \alpha _{i,i}\varvec{\Theta }_{s}\textbf{x}_{i} + \sum \limits _{j \in \mathcal {N}(v_i)} \alpha _{i,j}\varvec{\Theta }_{t}\textbf{x}_{j}\, \end{aligned}$$

where

4 $$\begin{aligned} \alpha _{i,j} = \frac{ \exp \left( \textbf{a}^{\top }\textrm{LeakyReLU}\left( \varvec{\Theta }_{s} \textbf{x}_i + \varvec{\Theta }_{t} \textbf{x}_j \right) \right) }{\sum _{k \in \mathcal {N}(v_i) \cup \{ v_i \}} \exp \left( \textbf{a}^{\top }\textrm{LeakyReLU}\left( \varvec{\Theta }_{s} \textbf{x}_i + \varvec{\Theta }_{t} \textbf{x}_k \right) \right) }\, \end{aligned}$$

$\textbf{a}$, $\varvec{\Theta }_{s}$, and $\varvec{\Theta }_{t}$ denote the learnable parameters for node $v_i$ and neighboring nodes $v_j$, respectively. The authors offer a special visualization that uses this information to enable users to get insight into the information propagation of the underlying GNN.

Their visualization aims to display the corresponding edge weights of each layer inside the GNN through transparency. In their case, GraphVQA’s GAT stacks five of these convolutions. Therefore, users can switch through the attention weights, $\alpha _{i,j}$, of each layer. This allows users to get an impression of how strongly certain edges contributed to updating the respective node embedding $x_i$ of node $v_i$.

#### Node attention weights

Because the authors deal with a graph classification problem, they need to aggregate the node embeddings into a single vector representation that captures the visual information of the system. In the implementation they are building on, this is referred to as graph gating mechanism. Essentially, they pool the node embeddings via a weighted sum into single vector $\textbf{x}_g$, as displayed in Eq. 5 (*N* refers to the number of nodes in the graph). Attention values $\alpha _{i}$ are computed between global language representation $\textbf{x}_l$ and node embeddings $\textbf{x}_i$, as shown in Eq. 6.

5 $$\begin{aligned} \textbf{x}_g = \sum \limits _{i}^{N} \alpha _{i} \textbf{x}_i\end{aligned}$$

6 $$\begin{aligned} \alpha _{i} = \frac{e^{\textbf{x}_l^{\top }\textbf{x}_i}}{\sum _{j}^{N}e^{\textbf{x}_l^{\top }\textbf{x}_j}} \end{aligned}$$

The authors hypothesize that attention values $\alpha _{i}$ carry important information to be visualized.

These attention scores are computed during the forward pass of the GNN, i.e., a score is specific to a given question-scene graph pair. Nodes with large attention values contribute more to the final result, and the opposite holds for nodes with low values. That is, the graph attention scores describe how much the final graph representation resembles each of the nodes before the final layers of the model are executed to select outputs. They can serve as an indication of the information the model chooses to amplify in the context of the question to then base its answer on. The proposed solution can visualize the node attention weights in the context of a question through the size of the nodes.

### Design and requirements

The authors main aim to design a visual analytics application for developers, that supports them in identifying strengths and weaknesses of their scene-graph based VQA system. This includes exploring false predictions and evaluating whether they are owing to the model, the underlying scene graph data, or ambiguity within the questions.

Researchers and developers are required to build their own scripts to analyze the data and gain qualitative insight into their model’s performance, which makes it difficult systematically search for patterns. Typically, this is done using common Python libraries such as NetworkX [40] and results in visualizations not tailored to scene graphs. This does not allow for an interactive exploration, which is helpful for graphs that contain too much information to visualize simultaneously.

Furthermore, the results for the dataset splits are not pre-computed and stored for easier access; however, they are computed on the fly, which makes a systematic analysis for error cases difficult. Additionally, altering and potentially fixing the input data to explore the behavioral changes of the model is difficult without a proper user interface, specifically for the visual modality. In the authors’ case, visualizing the scene graphs without the images might often be insufficient to explain issues during the model’s answer generation.

Hence, they elucidate the decision-making algorithm of GNNs to enable experts to efficiently find problematic input-prediction pairs. The tool could help developers debug the answer generation process and fix potential errors in the data sample, i.e., it should be possible to fix erroneous scenes via data curation. Consequently, the proposed approach is mainly designed for domain experts of graph-based VQA systems to evaluate and correct their models and data.

The authors collected experience with VQA [5] and a scene-graph-based VQA [41], which was useful to formulate a set of requirements that address the aforementioned issues.

- **R1** Visualize internal information of the model to increase understandability of the underlying mechanisms and enable to debug predictions (e.g., the node and edge attention values of the GNN should be examinable).
- **R2** Improve the prediction results through user inputs (data curation).
- **R3** Provide interaction to find, filter, and select relevant scenes for exploration and correction.
- **R4** Compatibility with state-of-the-art graph-based VQA models.
- **R5** Allow the system to be generalizable and expandable for other GNN-based approaches. 

To explore the data and identify erroneous scenes, the authors designed two different views: scene and evaluation browsers.

The scene browser enables users to visually explore the data, including a thumbnail image view with a brief overview of the underlying scene graph, i.e., displaying the number of nodes and relations within the graph. This supports users to quickly recognize problematic scenes. Complex visual scenes in images should be represented by comparatively similar complex scene graphs, which could be directly identified by looking at the number of nodes and relations. Additionally, through this view, it is fairly simple to search for images containing certain types of objects, attributes, or relations (R3).

The evaluation browser allows users to inspect the data on data sample level as well as the performance of a model on a given dataset split. Here, users can filter for certain questions and answers to retrieve corresponding data samples (R3). It is possible to directly search for false predictions that can be inspected to analyze the behavior of the model.

Both the scene and evaluation browsers allow the transition to a data sample level paired with an input-output visualization. This enables the users to interact with the inputs to the model. Here, the authors visualize the ground-truth scene graph on top of the raw input image, which is not used by the model. This supports users to evaluate the quality of the underlying scene graph, e.g., if the scene graphs capture all necessary information for a given question. Any question can be fed to the model via an input form to allow users to ask questions beyond the ones included in the dataset. Once a question is typed into the form, the tool replaces the ground-truth visualization with the information gathered during the decision-making process of the model to gain insight and extract evidence that explains the prediction (R1).

If the ground-truth scene graph is missing information or the annotated objects with their attributes and relations are incorrect, the tool offers the option to modify the existing data and add or delete falsely annotated elements (R2).

For the visualization of scene graphs, the authors selected a node-link diagram, which is often used by the community (e.g., refs. [1, 3]). A node-link diagram can be drawn on top of images (in contrast to an adjacency matrix), and the content of the image when nodes are placed on top of corresponding objects can be verified. Additionally, both nodes and edges can be used to encode additional information (R1).

It was important for the authors to reuse an existing graph-based VQA repository (R4) that they can built on to decrease implementation effort on the model’s side. Nevertheless, it was necessary to make the model exchangeable with other graph-based models (R5) to create a generic interface that allows for a fast integration with other repositories and models.

## Methods

Here, the authors present their interactive analysis system. In the “Locating scenes and instances” subsection, the different components of the visual analysis system to search for individual scenes (scene browser and evaluation browser) (R3) are explained. Additionally, details to explore ground truth visualizations (“Ground truth visualization” subsection) and inference-connected visualizations (“Inference-connected visualization” subsection) (R1) of individual scenes are introduced.

### Locating scenes and instances

An overview of the approach for examining the dataset is presented below. The goal is to support users in selecting individual scenes for further exploration (R3). This enables users to find flawed scenes and faulty predictions by the model that can then be traced down to being an issue of the model or the underlying input data. The latter can be directly tackled with the authors’ tool, as demonstrated on different use cases (“Use case” subsection). Furthermore, the authors evaluate their approach in a user study (“User study with experts” subsection).

Their visual analysis approach allows locating scenes in two distinct ways: (1) through a scene browser that enables the user to visually explore images and their corresponding scene graphs, and (2) an evaluation browser that focuses on the data sample level and provides features to search for desired properties.

#### Scene browser

**Fig. 3:**
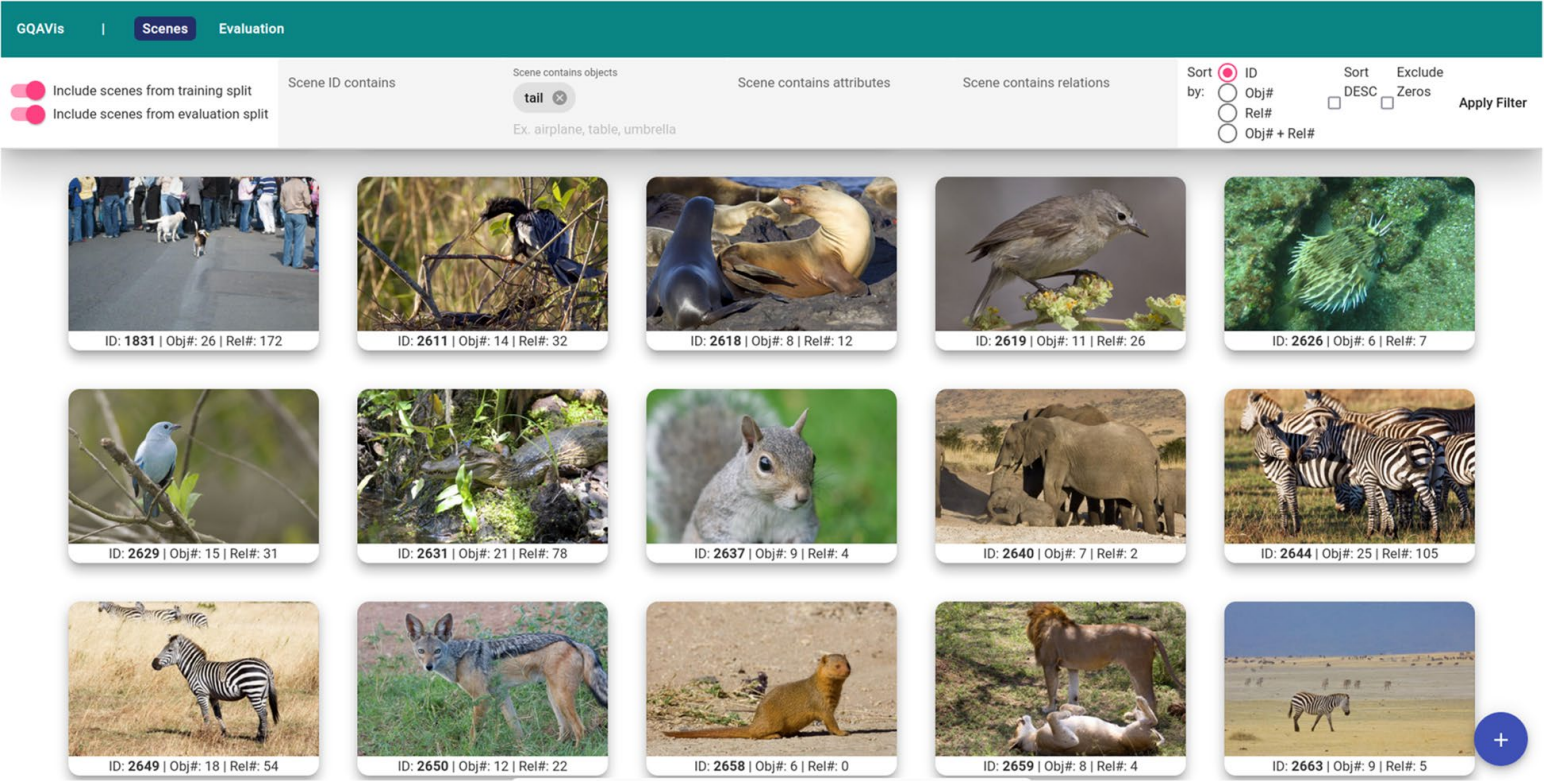
Scene browser shows a collection of different scenes visualized through their background images. In the bar at the top, several options for filtering the scenes are provided. Image thumbnails: GQA dataset [4] under Creative Commons CC BY 4.0

The scene browser, displayed in Fig. 3, offers an overview of all scenes in the dataset (training and evaluation data) in a grid layout. Each scene is presented as a card featuring a small preview of the corresponding image file, scene’s identifier, and number of nodes and edges in its corresponding scene graph. This querying approach is designed for a general exploration or if users are searching for specific types of scenes (R3), e.g., locating scenes containing certain types or numbers of objects. Additionally, it supports the assessment of the model’s familiarity with particular objects or concepts based on the number of scenes in which they appear on a coarse level. Scenes can be filtered based on scene identifiers, object names, attributes, or relation types. On a more fundamental level, scenes can be selected based on the dataset split they belong to, i.e., the training or the evaluation data. Sorting is available through scene identifiers, object counts, relation counts, or combined counts of objects and relations. Specially, a low number of objects and/or relations might indicate that important information in a scene graph is missing. Compared to the authors’ previous version [9], the scene browser presented in this work was extended. It provides an option for selecting ascending and descending sorting and to exclude empty scenes. These features offer support in finding incomplete scene graphs efficiently. Compared to the evaluation browser, the scene browser provides the possibility to explore scenes without a relation to the questions and answers of the dataset. Therefore, the performance of question-scene pairs using the prediction model is neglected. Instead, the raw scene data (images with information about their scene graphs) is presented. The images of scenes are shown to the user according to certain filter settings. It is possible to find scenes with a low or high number of elements or containing specific terms. The evaluation browser can be used to explore question-answer pairs given in the dataset.

#### Evaluation browser

**Fig. 4:**
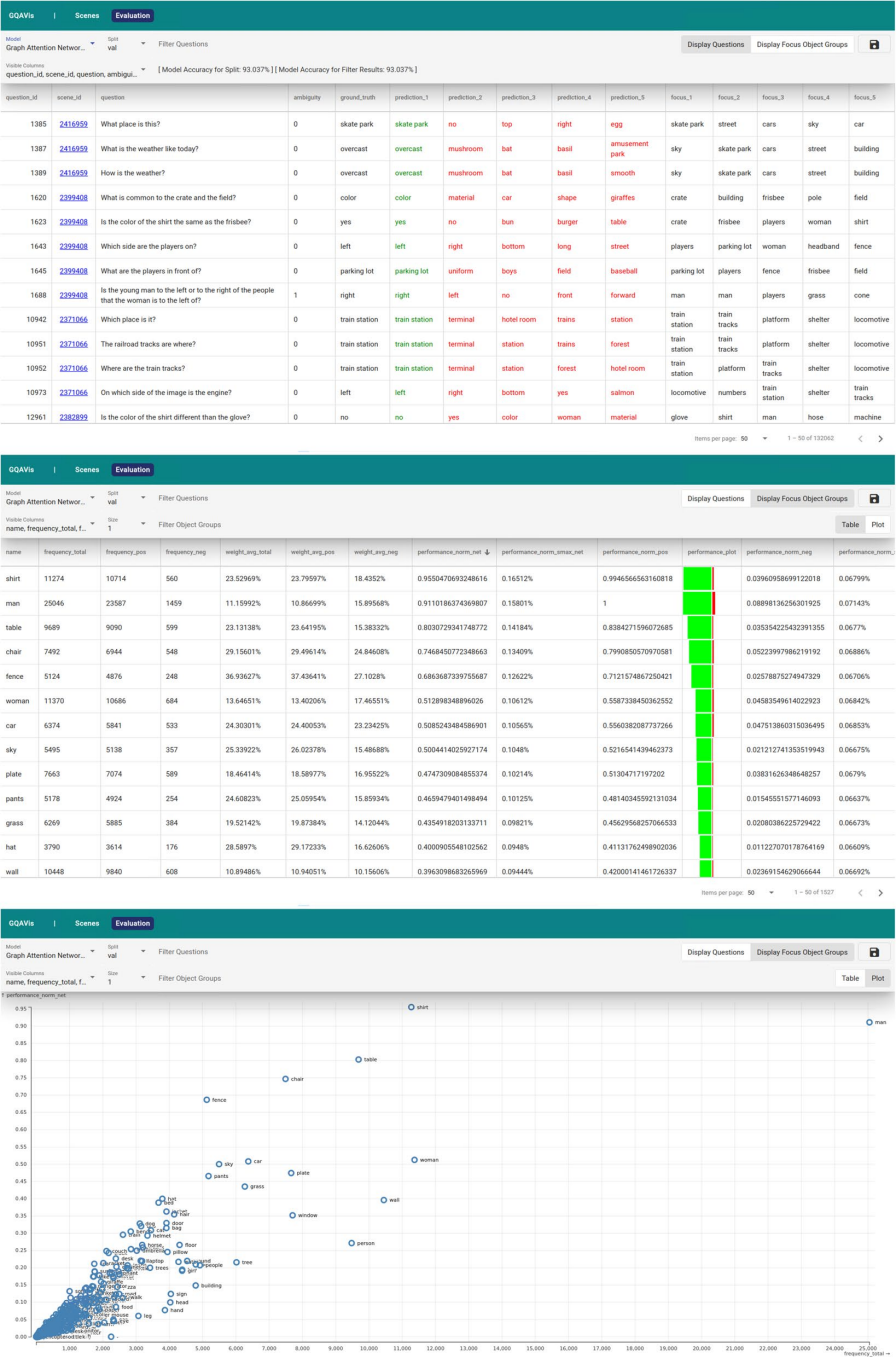
Example instance of the evaluation browser: For each question from the GQA dataset, the top five predictions and their prediction scores are displayed (top). Focus object groups, along with different performance values, are presented in a table (middle) and scatterplot (bottom)

The evaluation browser (Fig. 4) focuses on inference results and allows users to analyze the prediction results of all questions in the dataset (training and evaluation data). In contrast to the scene browser, the evaluation browser requires additional prerecorded data besides the ground truth data provided by the GQA dataset. Before querying is possible, the selected model has to process the questions. The top five generated predictions and their corresponding confidence scores are recorded, i.e., the probabilities from the softmax activation function, computed w.r.t., the predefined answer set. Each one is matched against the expected answer. Furthermore, the names of the five graph nodes with the highest gate weight (i.e., attention score) are recorded. The advantage of this querying approach over the scene browser is that it works based on data instances instead of scenes. Instead of searching for scenes that only represent one input modality, the user can locate question-scene pairs and retrieve the output of the model which provides more insights as well.

The evaluation browser provides two options for exploration (R3): (1) It is possible to explore questions and their prediction results in a table (Fig. 4 top). (2) The prediction results can be analyzed based on objects and object groups (consisting of objects with high attention scores) in either a table or a scatterplot (Fig. 4 middle and bottom).

The first view contains a table that consists of data sample entries. Each row displays different values: the question ID, content of the question, corresponding scene ID, a value for ambiguity, ground truth response, top five predictions along with their confidence scores, and largest five attention scores from the graph node aggregation. If one of the highest-ranked predicted answers is correct, it is highlighted in green, otherwise it is displayed in red. Ambiguity refers to an estimate of the potential uncertainty of the question and considers the number of objects in the scene graph the question may refer to by mistake.

There are different ways to filter for terms. The search can refer to the whole data or just individual columns. There can be exact matches (using the equal sign “=”), or a cell can contain the search term (using the colon sign “:”). Different search queries can be combined (using the and sign “&”).

This view helps users identify question-scene pairs that lead to faulty predictions. It also displays the model accuracy for the current filter results to support identifying cases where the model performs well or poorly. Users can select an associated scene to further explore model behavior using the authors’ inference-connected visualization (“Inference-connected visualization” subsection).

The second view is based on focus object groups. Objects included in such a group are taken from the set of graph nodes with the highest attention weights for all questions. Focus object groups are built from up to five of these terms that were included as one of the top five nodes for the same question. In the tabular form of this view (Fig. 4 middle), the columns contain information about the frequency of objects or object groups in the data in total, where the question has a correct prediction or a wrong prediction result. Additionally, different performance values are provided. In the scatterplot view (Fig. 4 bottom), the frequency of object groups is plotted against a performance value. In this view, it is possible to filter for objects and object groups to limit the presented information. Additionally, in this presentation, only the questions from the previously described filtering for questions are included. In summary, users may want to search for patterns in model responses using these kinds of queries.

### Ground truth visualization

In the authors’ ground truth visualization, the ground truth scene graph is drawn on top of the corresponding image (Fig. 1). A scene graph consists of objects represented as nodes with labels and edges showing the relationship between different objects. A connection between the scene graph and the image is visually established by coloring graph nodes based on the pixel data of their corresponding scene objects. Additionally, nodes are placed at the center of the respective bounding boxes of objects. Scene graphs can be large and create visual overlap; different design decisions were considered to minimize this. Interaction, such as filtering or hovering, helps explore the graphs. Users can modify the presented content by adding new elements (including the size of bounding boxes, labels and attributes of objects, and relations) or altering existing elements (R2).

Compared to the version of the authors’ tool used in Schäfer et al. [9], options for hiding the scene graph, adjusting the brightness of the scene image, adjusting node and edge size and edge color, and options to hide tool functionalities were added. The changes improve the visibility and visual presentation of the tool features.

#### Node colors

For node colors, the authors invoke a color depth reduction technique based on Median Cut [42]. This technique is more commonly applied to purposes related to color space compression. Given an object, they start by clipping the image to the object’s bounding box to determine a source domain. Operating in RGB color space, the color dimension featuring the widest value range is then chosen. Next, they sort the pixels of the source domain along this dimension. Finally, they cut the dimension in half along the median value of the source domain to produce two separate buckets. The dimension selection, sorting, and cutting steps can be repeated recursively, resulting in $2^d$ buckets. As they are only interested in two colors, they stop at this point. For the two resulting buckets, they generate representative colors by computing the average color across all pixels in a bucket. These colors are used to fill in the graph nodes.

Because representative colors are computed based on the average values of each pixel in a bucket, every pixel is represented equally in the resulting color and no information is discarded. This eliminates the risk of accidentally discarding characteristic colors of the source domain while remaining cost-effective and simple. However, representative strength of resulting colors decreases as the size and diversity of the source domain grow unless the bucket count is increased as well. Because the authors avoid this step, there is an inherent risk of under-representing interesting but rare colors, as would be the case for example with small details or objects contained within a large source domain. The approach therefore favors small source domains with one or two key characteristic shades. Highly representative colors for small bounding boxes capturing small objects are particularly useful, as graph nodes will naturally conceal more of the relevant parts of the object in the underlying image if the object is small yet sufficiently important to be represented individually in the graph.

#### Reducing visual overlap

**Fig. 5:**
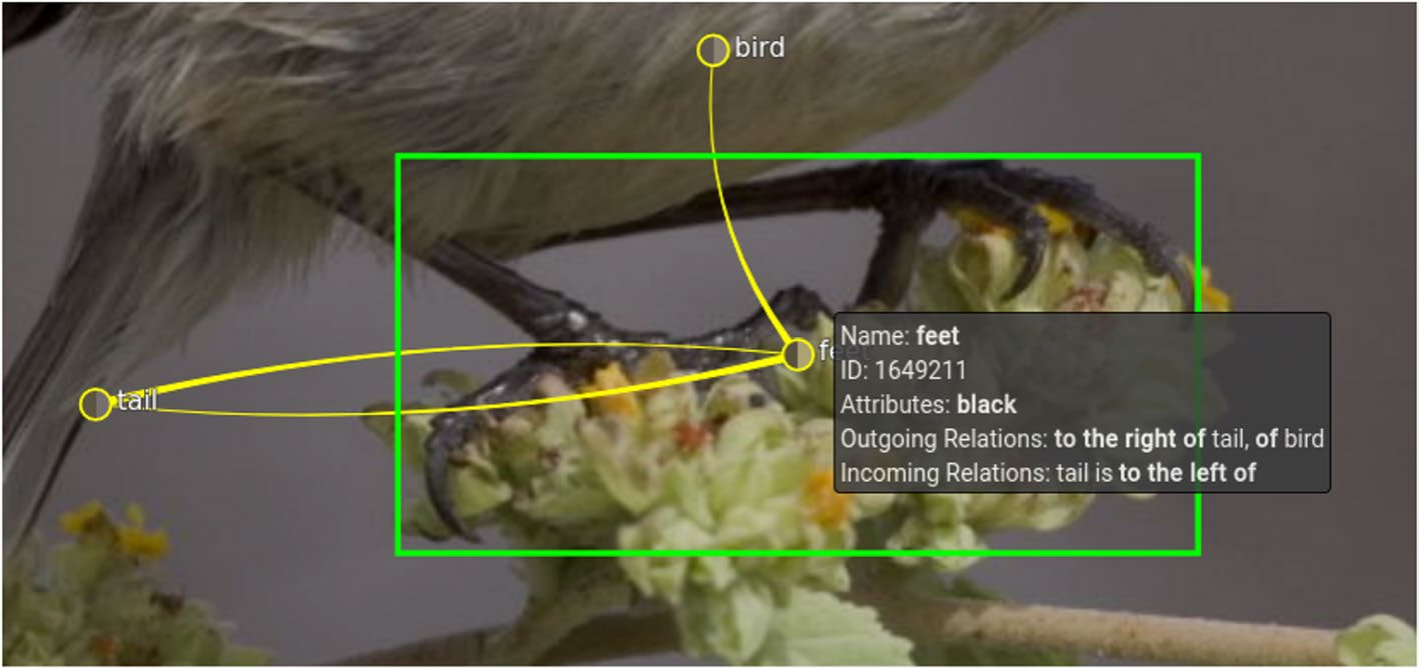
For a selected node from the scene graph, the bounding box is shown in green, and further information about the node is provided as a tooltip. Background image: GQA dataset [4] (scene 2619) under Creative Commons CC BY 4.0

As scene complexity increases and scene graphs grow, the visual overlap between graph elements tends to increase, thus leading to a more cluttered display. This has an undesirable effect on the readability of the graph. The authors mitigate this effect by making some careful design decisions for the visual representation of objects (sections of the image represented with graph nodes) and relations (graph edges). The source dataset describes objects using bounding boxes that map onto the image. Because most scenes contain numerous objects of varying sizes and spatial arrangements, overlap between bounding boxes is very common. To allow for an intuitive visual mapping between graph and image contents, they set up graph nodes as centroids of object bounding boxes (Fig. 5). This introduces a constraint: nodes have fixed positions. Techniques based on repositioning graph nodes, such as force-guided layouts that could be used to automatically find presentations with small overlaps, are not applicable. Instead, the authors follow these basic principles: (1) Object bounding boxes are hidden by default. (2) Smaller circles are drawn on top of larger ones to prevent complete occlusion.

As relations among objects are directed, it is the norm that two graph nodes representing two different objects with relations between each other are connected via at least two edges. The model they build on synthesizes counter-relations deems missing to support two-way message passing between nodes in its GNN layers. Both relations between two nodes are symmetric and belong to the same relatedness while possibly differing in the ordering. Therefore, they visualize each relation individually based on the following principle: Relations are drawn as parabolas with vertices offset to the left of the center based on a predetermined relation ranking. This principle allows us to avoid overlap for both symmetric and parallel relations between two objects. Furthermore, the curvature direction makes it easy to perceive the direction of a link. Graph readability is further improved through filtering mechanisms and dynamic highlighting or masking of graph components and sub-graphs following user interaction. To differentiate between relation types in the visualizations, the authors use green for synthesized counter-relations and yellow for relations natively present in the scene graph.

#### Interactive display of information

The initial state of the ground truth visualization is optimized to provide an overview of the scene. The authors extend the capabilities by introducing a disjunctive filtering mechanism. Any object matching at least one of the filters is displayed, whereas the remaining structure is hidden. Filters can target object names, object attributes, and labels of relations. Options on top of filters allow a selection of elements to be displayed.

Besides filter-based masking of graph elements, direct user interaction further narrows down the structural information displayed at any time. Hovering over a graph object with the mouse cursor makes the object bounding box visible. Furthermore, the graph visualization is reduced to the local neighborhood of the selected node based on user configuration. Hovering over relations shows only the relation itself, the two connected object nodes, and both of their bounding boxes. Tooltips contain additional information on the element that is subject to hovering (Fig. 5). If the element is an object, the tooltip lists textual descriptions of the object’s incoming and outgoing relations and the object’s name and attributes. For relations, only a textual description of the relation and the names of the two connected objects are presented.

A sidebar component contains an overview of every element currently visible in the graph visualization (Fig. 1E). For each entry, the name/description and attributes of the element, in the case of objects, are featured in a list. To aid with the identification and correlation of list entries with graph elements, each element in the graph can be clicked to highlight its corresponding list element and vice versa.

#### Assisted editing of the ground truth

Model predictions are generated from two inputs: a natural language question and a scene graph. Beyond topological information in the graph structure, element labels are important as they are tokenized and later used to initialize different parts of the NN. For example, graph node features are initialized using GloVe 300d vector embeddings [43] of the node’s name and attributes. As these are pre-trained, previously unseen tokens carry no particular semantic value. To assist users with generating rich and meaningful model input, all user-interface input elements offer autocomplete suggestions sourced from a dictionary of vocabulary known to the model.

### Inference-connected visualization

**Fig. 6:**
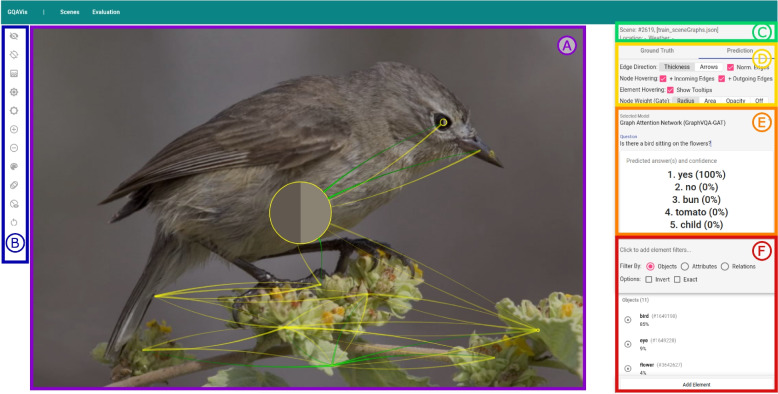
The prediction view shows the response to an input query (**E**) along with the scene graph weights for each node and edge the GraphVQA-GAT model computed (**A**). The graph gate weights after the final graph convolution are used for node sizes and edge weights are used for the transparency of relations. Additionally, there are different options for the visibility of the image and scene graph on the left (**B**). The panel on the right provides distinct information and settings: meta-information about the current scene (**C**), settings for the appearance of the scene graph (**D**), a question area with five prediction results (**E**), and a list of all objects and relations of the scene graph with weights and filter options (**F**). Background image: GQA dataset [4] (scene 2619) under Creative Commons CC BY 4.0

The authors’ inference-connected visualization mode (Fig. 6) builds upon the ground truth visualization by introducing additional information to be presented on top of the ground truth graph. Certain annotations, such as node and edge labels, take on a less prominent role to create space for visualizing internal states (R1) of the GNN during the forward pass. Tool-tips connected to elements in the graph contain further contextual information about the element, e.g., the node’s identifier and corresponding attributes, or relation types.

#### Assisted model inference

Similar to their assisted editing of the ground truth, the question input field (Fig. 1D) is augmented with suggestions generated from known tokens included in the model’s vocabulary. This enables users to carefully select semantically rich tokens while posing arbitrary questions. The inputs are preprocessed by an adapter component that bridges most model specifics to a common interface. During model execution, various internal model states are recorded to drive visualizations later on. For the GraphVQA integration, the authors record the global node attention scores (graph gate weights) and the attention head weights of edge attention $\alpha$ during all five convolutions of the GNN layers. Model outputs in the form of short answer logits are recorded as well. The authors calculate the softmax over all classified output categories’ prediction scores to arrive at a displayable model confidence score for each possible answer to increase interpretability (compared to arbitrary values). The labels of the five highest-ranking categories and their confidence scores are displayed. Additional predictions are provided beyond the top prediction to enable users to interpret the output in more depth.

In the visualization of the scene graph (R1), the weight share of each edge is translated directly to opacity. The sum of edge weight shares and the portion that a node’s own previous value contributes is 1. Because the weight factor of nodes is often reasonably large compared to the factors of the edges, it is common for all edges in the graph to have a very low resulting opacity. Therefore, the authors added an option to additionally normalize all edges globally such that the edge with the largest recorded weight (per step) is opaque while the rest is scaled accordingly. The node sizes encode the graph gate weights, allowing users to find objects with a high or low focus attained by the model. More details about the corresponding values are presented in the “Graph neural network visualization” subsection. In Fig. 6, the graph gates and edge attention are visualized for an example inference on the GraphVQA-GAT model.

## Results and Discussion

The authors demonstrate typical use cases of how their system can be utilized to explore (R3), understand (R1), and correct predictions (R2) generated with the trained model. Thereafter, the results of a quantitative evaluation analyzing the relation of model internals to the inputs and outputs are reported. In addition, they present the results of a user study conducted to verify the usefulness of the proposed approach.

Here, an instance of the Graph-VQA-GAT model trained is used. With an accuracy of around 93%, the authors’ model parameters perform similarly but not quite as well as the parameters trained by Liang et al. [3]. In the authors’ system, it is possible to explore the training and evaluation split (images, scene graphs, and questions) of the GQA dataset [4]. The data they use consists of around 86,000 images (around 11,000 in the evaluation set and 75,000 in the training set) and more than 1 million questions (132,062 in the evaluation set and 943,000 in the training set).

### Use case

Their visual analysis system can be used to examine (R3) and interpret (R1) predictions paired with internal information of the GNN to adjust the input data to generate more accurate predictions (R2).

Poorly labeled scenes often do not create satisfying predictions by the model. In their tool, finding such scenes (R3), exploring them (R1), and improving their scene graphs (R2) for better performance play a central role. This improves the underlying dataset through annotation. However, false predictions generated by the model occur because of a faulty scene graph, e.g., when specific types of questions, objects, attributes, or relations contribute to false predictions. Identifying such problems with their system is possible; however, improving the performance of the model for these cases is more challenging. The authors would be required to fine-tune the model to these aspects by feeding improved or new training material into the model to improve the model’s performance in some instances. Therefore, in the examples below, the authors focus on finding (R3) and understanding problems (R1) in the prediction owing to the poor quality of scene graphs, improving the scene graphs (R3), and increasing the performance of the predictions (R3). Additionally, they present examples of correct and incorrect predictions, how a user can gain insights into the internal mechanisms (R1) of the model, as well as the possibility of adding new scenes to the data set.

In their visual exploration, the authors noticed that the edge weights often did not provide helpful or meaningful internal information about the model to the user. This observation was also verified in their quantitative evaluation (the “Quantitative evaluation” subsection). Sometimes, one of the edge attention convolutions showed a sense-making relationship between higher weights of relevant edges for answering the current question. However, the edge attention convolution and whether meaningful information was available varied for every image and question. Therefore, using these edges in the analysis is possible; however, it does not always help to better understand the model’s internals. In the demonstration below, the authors do usually not mention the edge weights but focus on the graph gate weights represented as node sizes.

#### Finding poorly labeled scenes

Data curation (R2) is essential for a high-quality dataset. It includes the dataset being constantly maintained and improved. In this context, the authors attempt to refine and enhance their dataset using the visual analysis approach. As the first step, they need support finding poor scenes (R3) that may require human corrections (R2). Selecting arbitrary scenes of the 86,000 available images (the images from the training and evaluation set) and deciding the scenes that require correction could be tedious and time-consuming. Therefore, the scene and evaluation browser can be utilized to find such scenes.

##### Scene browser

**Fig. 7:**
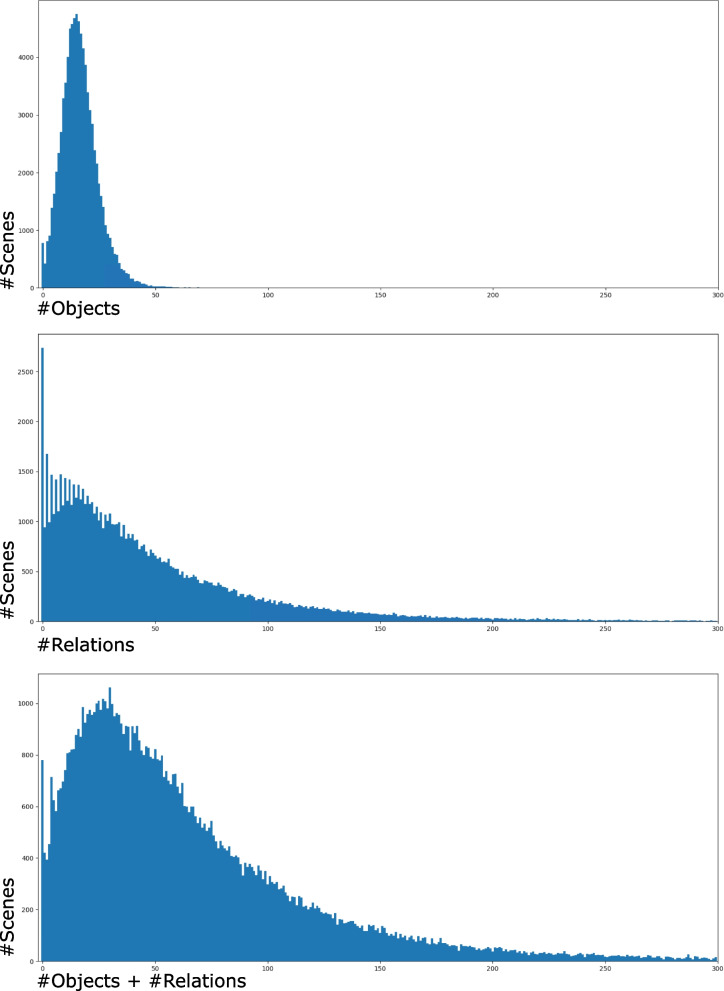
Number of objects and/or relations compared to the frequency of their occurrence in scenes. The *y*-axis represents the number of scenes and the *x*-axis represents the number of objects (top), relations (middle), and objects plus relations (bottom)

In the scene browser (Fig. 3), different filters can be used to search for specific scenes (R3). In addition to searching for specific labels in the scene graphs, the data can be sorted by the number of objects or relations in a scene. Sorting the scenes by either the number of objects or relations, the authors identified many scenes without scene graphs or just a low number of objects and relations. In extreme cases, neither objects nor relations were defined. Therefore, such scenes look all the same to their model in the prediction process, and it is clear that these scenes cannot create correct answers for most questions and need correction. They looked at the general distribution of the number of objects and relations in the scenes of the given dataset (Fig. 7). The results showed that the number of scenes with no objects or relations is considerably large (781 scenes have neither objects nor relations and 2738 scenes have no relations). Many scenes have a low number of objects/relations (421 only have one object and 943 have just one relation). Although a low number of objects and relations can indicate an image of little content, their verification of some of these scenes showed that there were important features of the images in the scene graphs missing. Figure 7 also shows that common to most scenes is that they are represented by approximately 15 objects and 10 to 20 relations. Using the authors’ approach, it is possible to select a scene, create a new scene graph, or add/correct objects, relations, and attributes of objects.

In addition to finding scenes with a low number of labels, the quality of specific labels can be verified with the help of the scene browser. When filtering for a specific term (e.g., for objects), the user receives a collection of the scenes that should contain this term. However, the authors noticed images where the relevant object was not visible. When looking more carefully at the individual scenes, the objects were sometimes still available but extremely small. However, there were other scenes where the objects were not visible in the image, and the label was set for a different object. Such scenes also require correction.

##### Evaluation browser

The evaluation browser has two different views. Using the table for questions (Fig. 4 top), users can sort for questions and corresponding scenes that created wrong predictions (using, e.g., the column *correct_1* that contains either *true* or *false* depending on a correct or incorrect first prediction result) or prediction results with low confidence (using the column *confidence_1* that contains a numerical value for the confidence). Both can be used to identify (R3) the corresponding scenes that may need an improved scene graph for better predictions (R2). The authors present more details about the different types of errors later.

Exploring specific scenes is also possible for the evaluation browser. Users can enter a scene ID (using *scene_id=*), and all questions for this scene are shown along with the model accuracy for the current selection of the provided questions. Similarly, filtering for specific terms in questions and (ground truth or predicted) answers and getting the model accuracy for the corresponding questions is possible. Additionally, results can be sorted by ambiguity. Questions with high ambiguity could be wrongly answered because the model could give a wrong object the highest weight for answering the question.

Another way to use the evaluation browser is to look for objects that have a low performance. In the form of a table (Fig. 4 middle) or scatterplot (Fig. 4 bottom), objects or object groups can be identified with low prediction quality. In the table, it is possible to sort by the number of questions that created the wrong prediction with the given object (group) or had a low-performance value. In the scatterplot, the relation between the frequency of objects or object groups and the performance value are visible. Additionally, for both representations, the data can be filtered, e.g., by certain terms for the object groups, and the performance and content of the questions that should be considered.

The next step would be to use such terms in the scene browser to search for the scenes and verify them.

In the tables of the evaluation browser, when a user wants to inspect the corresponding scene of a question, it is possible to immediately jump to the linked scene.

#### Exploring scene graphs

The scene graphs can be explored by inspecting the ground truth representation or the result shown after posing a question (R1). Here, node gate weights and edge attention are presented.

##### Ground truth scene

When exploring a ground truth scene with a scene graph (see the “Ground truth visualization” subsection), it is sometimes immediately visible how well a prediction might perform for some question. Without deeper exploration, it can be seen if most of the image content is represented as objects and if relations connect these objects. Sometimes, there are insufficient objects and only questions for specific objects can be answered correctly (see the “Missing object” subsection). Because labels for objects can be shown, it is apparent to what part of the image nodes belong. The authors observed that relations were often available, but more relations would be required to process a scene fully for many questions. Just parts of a scene graph were often connected (see the “Missing relation” subsection). Further interaction through hovering over objects or relations and inspecting the tooltips shows if they also have (correct) properties or attributes.

##### Posing a question

After posing a question, on the right side of the window (Fig. 1D), it is visible what the model predicts along with its confidence and four following best prediction results. Additionally, the scene graph shows internal information (R1) of the underlying model encoded as node size and edge transparency (see the “Inference-connected visualization” subsection for more details). Questions often refer to objects, their relation, or properties of objects/relations. Typical questions ask for the location of an object, possibly relative to other objects. Others refer to the visibility of particular objects including their properties (e.g., color, material, or size) or what someone/something is doing/the current state. Finally, there are yes/no questions that may refer to the previously mentioned aspects. Depending on the type of question, an incorrect prediction might be caused by the corresponding elements of the scene graph or owing to pre-trained information being available in the model.

**Fig. 8:**
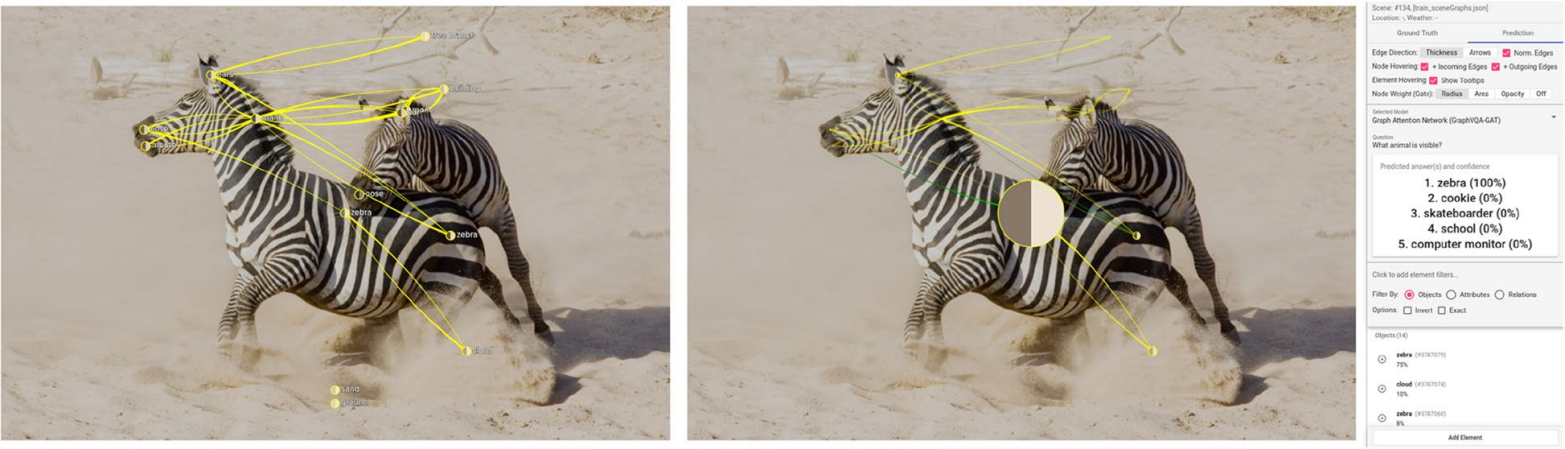
Correct prediction: After posing the question “What animal is visible?” the model answers correctly with “zebra” (100%). The node of the zebra in the front also has the highest gate weight (represented as node size). The ground truth scene is visible on the left, and the prediction view for edge attention convolution one is on the right. Background image: GQA dataset [4] (scene 134) under Creative Commons CC BY 4.0

**Fig. 9:**
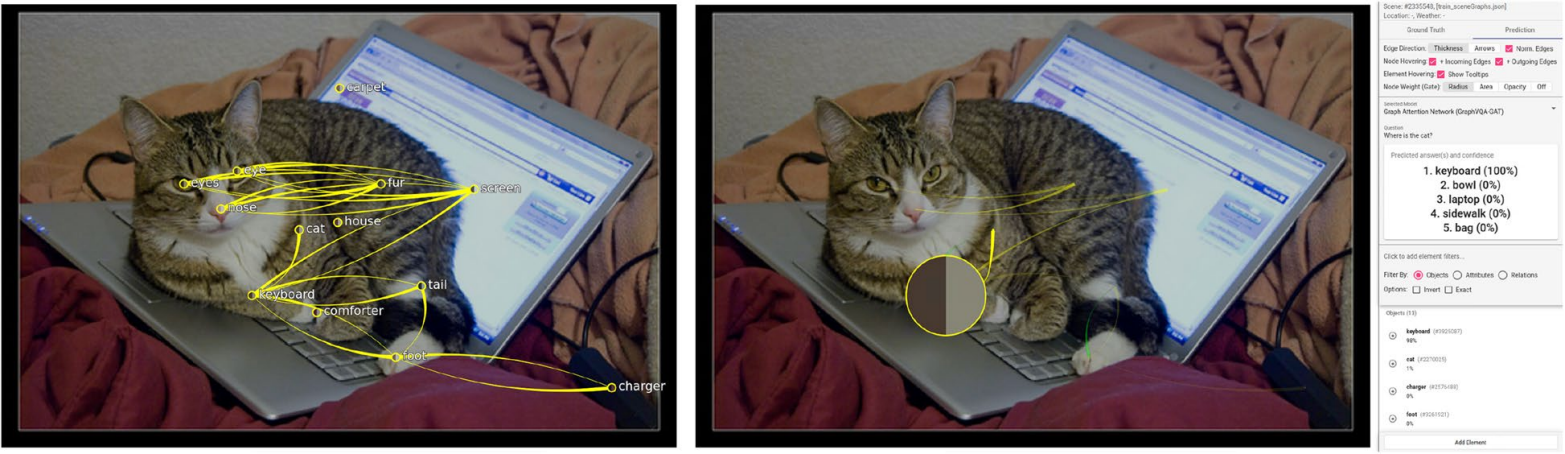
Correct prediction: After posing the question “Where is the cat?” the model answers with 100% certainty “keyboard.” The node “keyboard” has the highest gate weight followed by the node “cat.” In the visualizations on the right, the transparency of the edges shows the results for edge attention convolution four. Here, the relation between the “cat” and the “keyboard” has the highest weight. Background image: GQA dataset [4] (scene 2335548) under Creative Commons CC BY 4.0

**Correct prediction** – Figure 8 shows an example of a correct prediction. The question was “What animal is visible?” The answer was correct (“zebra”, 100%). Additionally, the relevant object “zebra” has the highest gate weight. To answer this question, the model needs internal information about what an animal is and connects its knowledge to the availability of objects in the scene. The edges do not play an important role in answering this question. In another example (Fig. 9), the model correctly answers the question “Where is the cat?” with “keyboard.” The relevant object for answering the question has the highest weight: “keyboard.” In this example, the authors also looked at the weights of the edge attention convolutions. In edge attention convolution four, the connection between the “cat ” and the “keyboard” has the highest weight, verifying that this edge was relevant for answering the question. In the other convolutions, there was no significant difference in the weights of the different edges. These are typical examples of correct predictions. If the relevant objects for answering a question do not have large gate weights, the model might be rather uncertain with its prediction.

**Incorrect prediction** – For incorrect predictions, there can be different sources of errors. In addition to a poorly trained model, the scene graphs could contain errors or be incomplete. The sources of errors and ways to improve them are presented in more detail below.

##### Sources of errors and their correction

In their evaluation, the authors noticed different types of prediction errors that can be created with the model and required different corrections in the scene graphs. The different sources of errors can be categorized as follows:

- Missing scene graph
- Missing object
- Missing attribute of an object
- Missing relation
- Incorrect label (of an object/relation/attribute)
- Ghost object
- Multiple correct (but unexpected) answers

The different problems in the scene graph are described below, and examples of how they can be solved are presented.

**Fig. 10:**
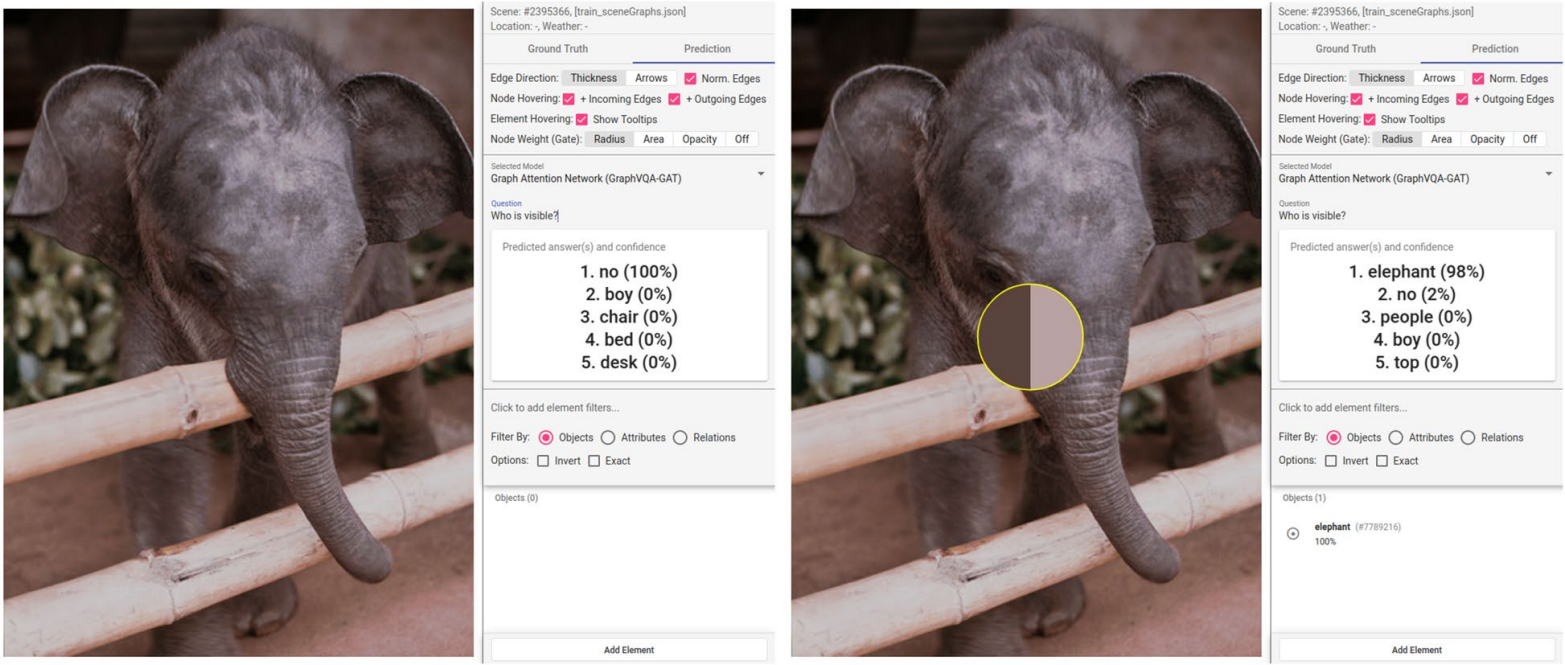
Incorrect prediction: After posing several questions about the scene’s content, the model answers incorrectly. This is because no scene graph was defined in the dataset. Adding the node “elephant” generates a better performance for questions such as “Who is visible?” The incorrect prediction is visible on the left, and the corrected result is on the right. Background image: GQA dataset [4] (scene 2395366) under Creative Commons CC BY 4.0

**Missing scene graph** – If no scene graph is available for an image, it is evident that no correct prediction about the content of an image can be performed. The authors noticed that for some of such scenes, no questions were available in the dataset, which could indicate that these scenes have low performance. They posed different questions to such a scene to grasp what general information of the model is available for the answer generation. An example scene is visible in Fig. 10 showing an elephant. Many questions received quite acceptable answers considering that there was no scene graph available but still incorrect for the respective images. Questions about the visual content such as “What is visible?” (“no”, 90%), “Who is visible?” (“no”, 100%), “What can you see?” (“no”, 100%) received with high percentages the answer “no” because nothing is available. However, a similar question, “What is in this image?” (“chair”, 60%) or “What is in this picture?” (“chair”, 100%) had the answer “chair” with high percentages. For such questions, the model uses general information from the training. Verification in the evaluation browser (Fig. 4 bottom) shows that “chair” is a common object, and its prediction quality is very good. Users can now manually create a new scene graph to provide relevant information about the image content. This can be done by adding new objects with attributes and relations between objects. Adding just one node to this scene (“elephant”) creates more reasonable results to many questions such as “Is there an elephant?” (“yes”, 100%), or “Who is visible?” (“elephant”, 98%).

**Fig. 11:**
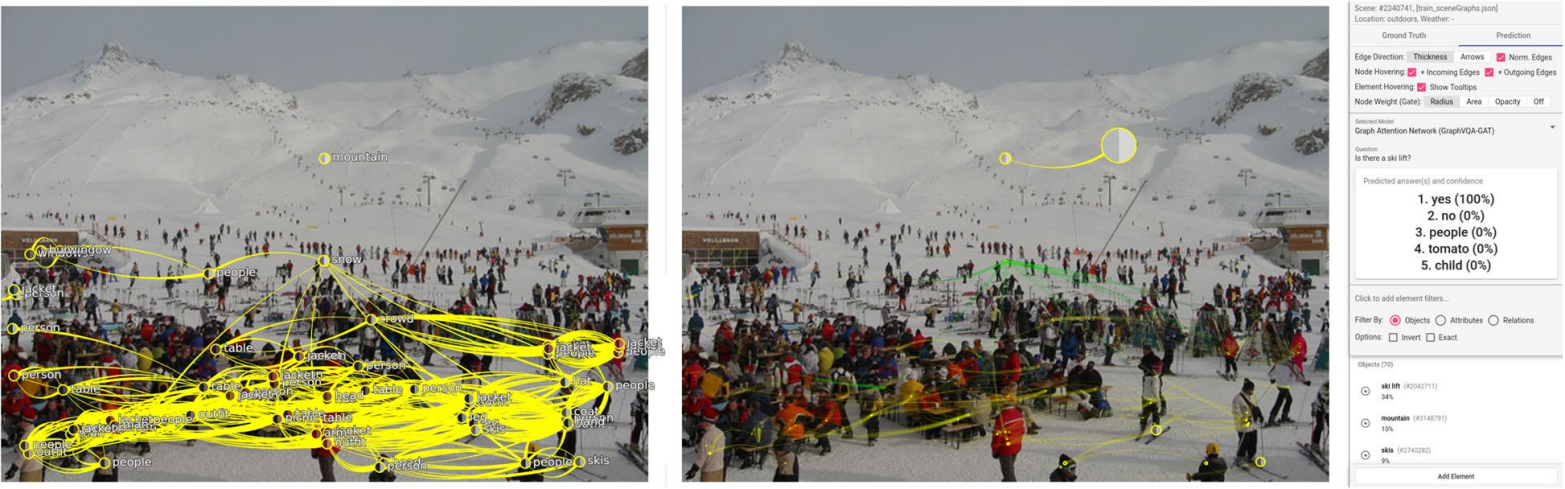
Incorrect prediction: The left side shows the ground truth scene graph and the right side shows the result after the prediction. After posing the question “Is there a ski lift?” the model answers with 100% certainty “no” because no object for “ski lift” is available. After adding a corresponding object with relation to the mountain, the prediction is correct; this is shown on the right for edge attention convolution four. Background image: GQA dataset [4] (scene 2340741) under Creative Commons CC BY 4.0

**Missing object** – Missing objects often result in wrong predictions when subject to the question. If the question is unrelated to a missing object, it may not generate wrong predictions. The previous example was already such a case. However, many objects are often defined for a scene; however, some are still missing. An example is shown in Fig. 11, a scene already containing 69 objects and 592 relations. The authors asked: “Is there a ski lift?” The answer was incorrect. The obvious answer would have been “yes;” however, the model answered with “no” (100%). Inspecting the scene shows no node is available for “ski lift.” While an object could be available in the prediction mode but very small, verification in the list of objects (on the right side) or the ground truth scene graph indicates no such object. After adding it to the scene graph (along with a relation), the authors’ model performs a correct prediction. In contrast, the question “Where is the ski lift?” gets the answer “snow” (100%), and the object “snow” has the highest gate weight. Although this is correct, a different image without a “ski lift” but “snow” most certainly would have received the same answer because general information of the model was used. Adding the object “ski lift” in relation to the mountain could provide the expected and correct prediction result (“mountain”, 100%).

**Fig. 12:**
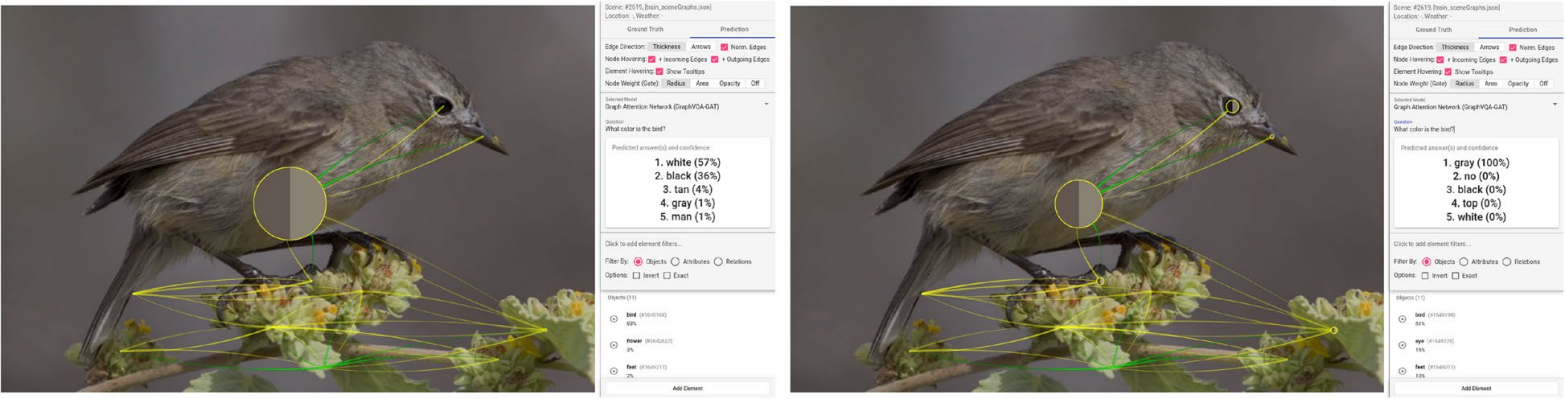
Incorrect prediction: In this example, the authors asked: “What color is the bird?” (Left) The model answers incorrectly with “white.” (Right) The answer is correct after adding the attribute “gray” to the bird. The prediction views show edge attention convolution one. Background image: GQA dataset [4] (scene 2619) under Creative Commons CC BY 4.0

**Missing attribute of an object** – Node labels for objects alone do not always provide all the information needed about them. Additional information such as the appearance (e.g., color) is essential. Such missing attributes often result in bad predictions. While the scene graphs look complete and suggest that the correct objects have the highest weights, the answers are still incorrect. Similarly, the authors use the example in Fig. 8 showing the zebras. This time, they asked: “Where are the zebras?” The model answered incorrectly with “sky” (100%). The weights in the scene graph seem correct at first sight. The highest weight has the object “cloud.” However, the “cloud” has no attributes, and their model assumes from its general knowledge that clouds must be in the “sky.” Adding the attribute “sand” to the “cloud” results in a correct prediction. Now, the model answers with “sand” (100%). Another example is shown in Fig. 12. They asked: “What color is the bird?” The model assumes that it is “white” (57%). Additionally, the node “bird” has the highest weight. However, it does not have any attributes. After adding the attribute “gray” to the object, the answer of the model is also correct (“gray”, 100%).

**Fig. 13:**
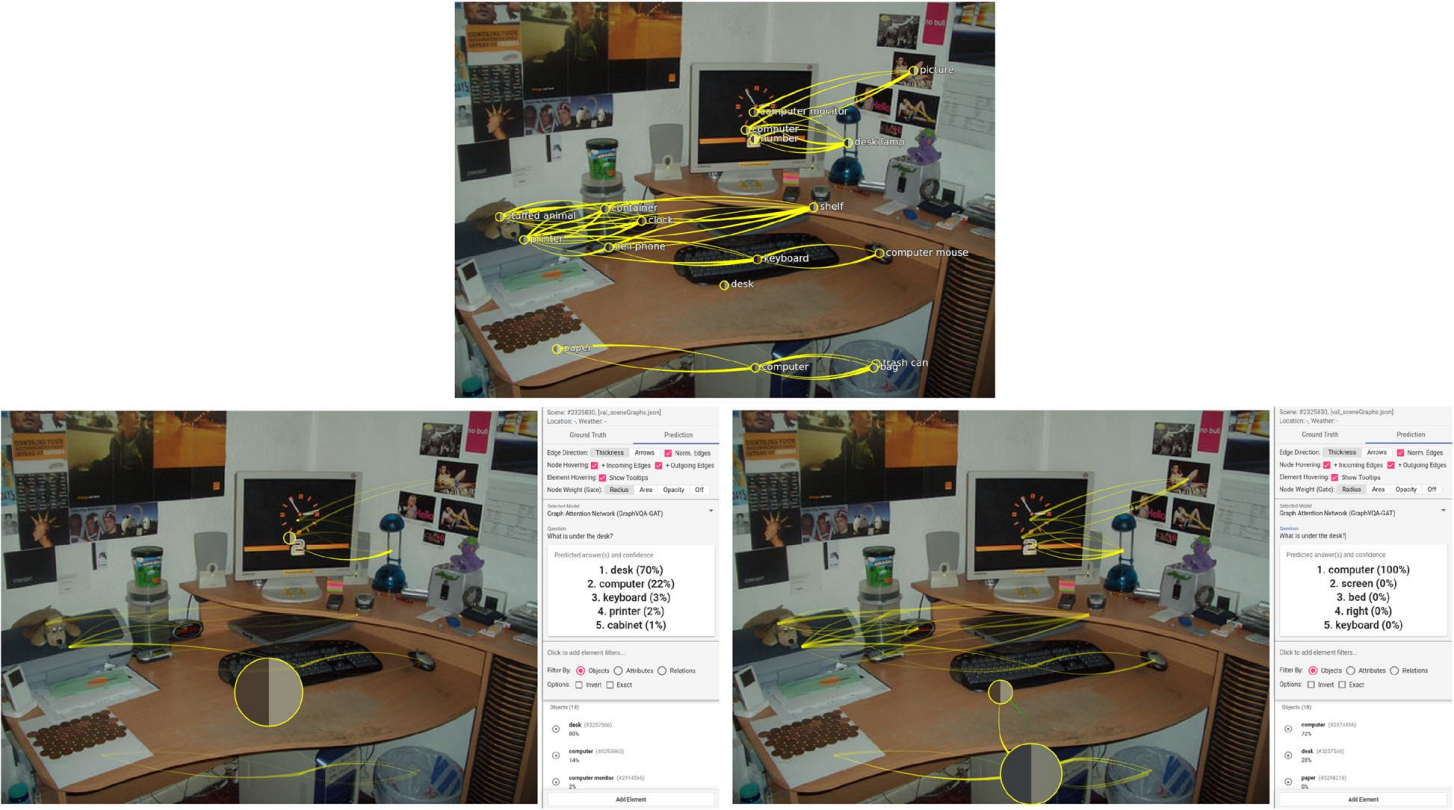
Incorrect prediction: Multiple clusters are visible in the ground truth scene graph (top); Asking the question “What is under the desk?” generated the wrong answer (“desk”) (bottom left); The correct prediction was made after adding a relation between the “desk” and the “computer” under the desk (bottom right). The prediction views show edge attention convolution two. Background image: GQA dataset [4] (scene 2325830) under Creative Commons CC BY 4.0

**Missing relation** – Another source for poor predictions is missing relations. Only if the connections are available along with meaningful labels questions about the relationship between different objects (such as relative positions or if something is otherwise related, e.g., someone is holding/wearing something) can be answered. Often, many objects are defined in the scene graph, but connections between objects in the picture are missing. In their evaluation, the authors often noticed that different groups of objects or single objects were not connected. Positional questions about these groups could then not be answered correctly. Many relations would be required to provide all the information visible in a scene. In the best case, a fully connected graph encodes all information. Such a graph is seldom available; debugging such a graph is also very difficult owing to a large amount of information and visual overplotting. Additionally, edges would contain unimportant information. When the authors use the example in Fig. 8 and ask the question “Where are the zebras?” they could also expect the answer “ground.” This is an object available in the scene graph. However, there is no connection between this object and the remaining objects. Adding the relation “on” between the “zebra” and the “ground” generates the expected prediction. Another example, with multiple clusters of objects, is visible in Fig. 13. Here, they ask: “What is under the desk?” An expected answer would be “computer.” However, the model answers with “desk” (70%), and the object “desk” has the highest gate weight. Investigating the ground truth scene graph shows that the part on top of the desk is not connected to the area under the desk. Simply adding a relation between “computer” and “desk” with the label “under” generates the correct result with 100% certainty, and the “computer” has now the highest gate weight followed by the “desk.”

**Fig. 14:**
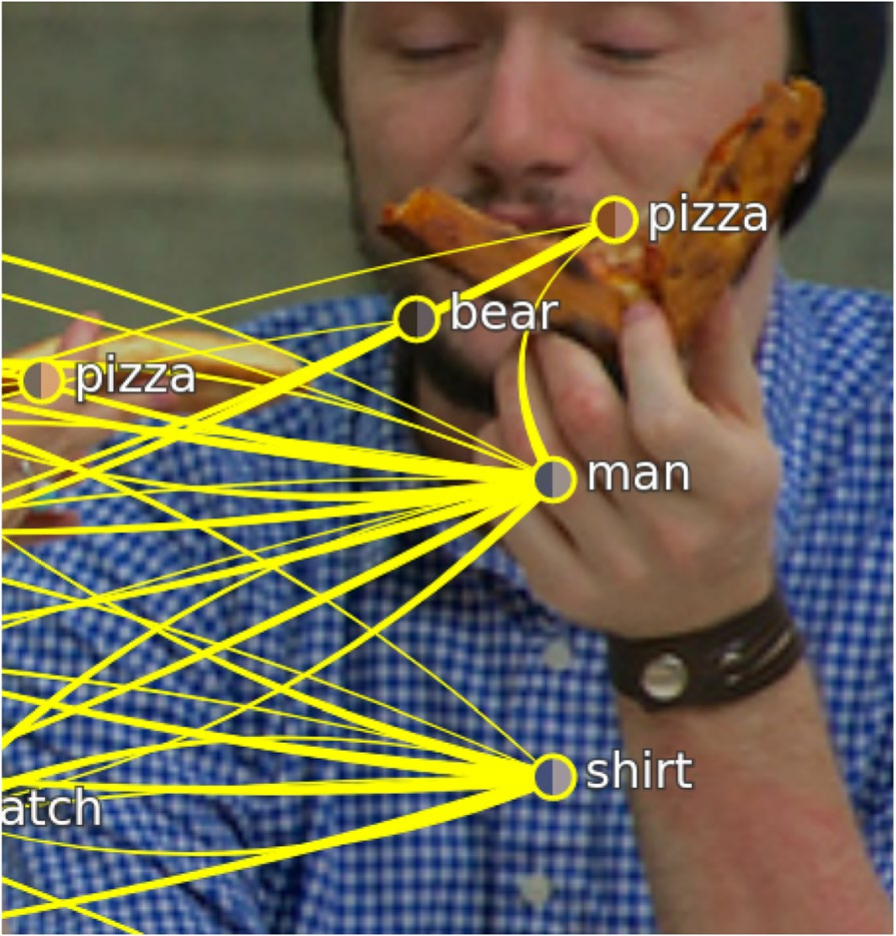
Erroneous scene graph: Excerpt of a scene showing a man with a “beard.” In the scene graph, the “beard” was incorrectly labeled as “bear.” Background image: GQA dataset [4] (scene 2351646) under Creative Commons CC BY 4.0

**Fig. 15:**
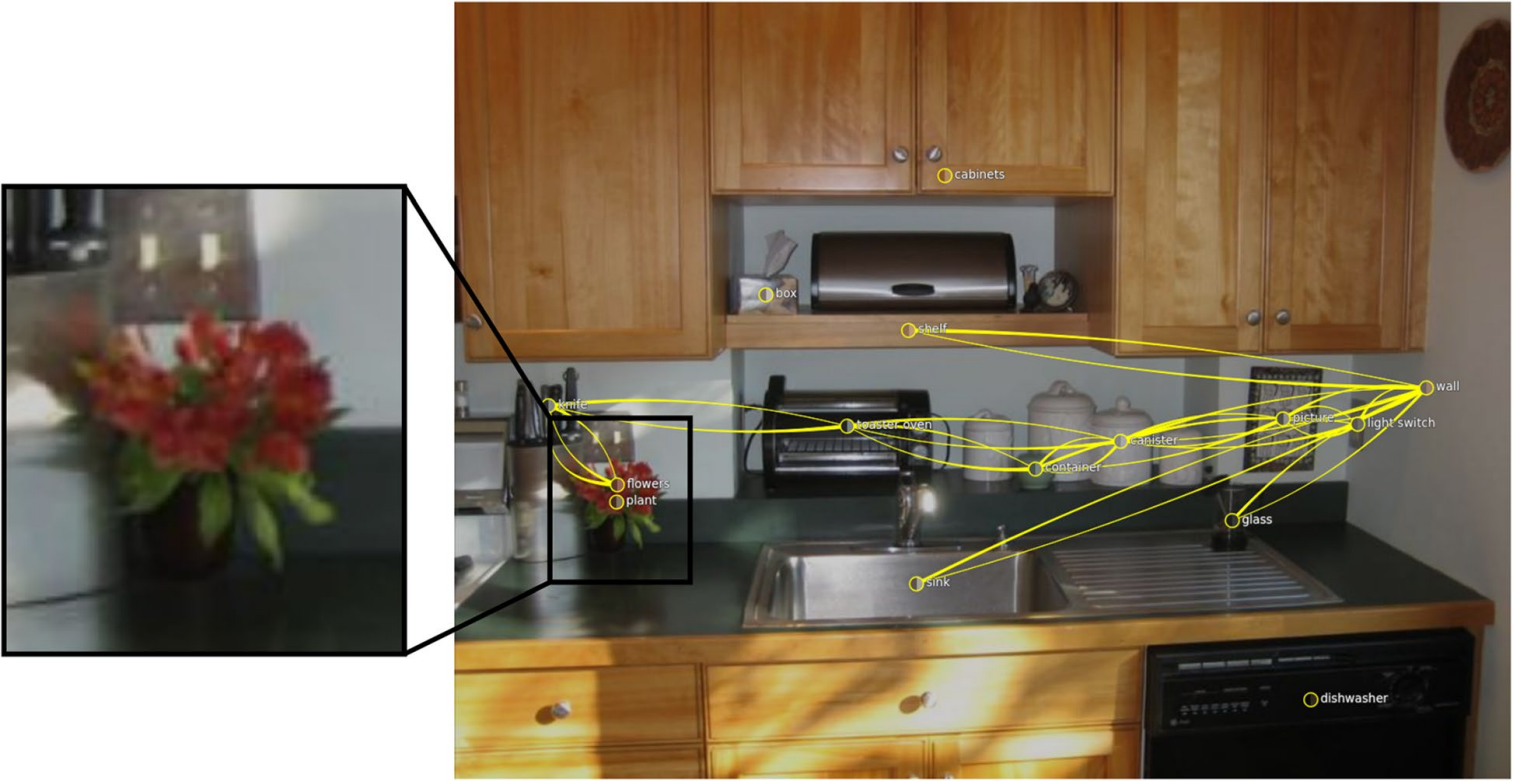
Incorrect prediction: When the authors ask the question “What color are the flowers?” the model answers with “orange.” Changing the attribute of “flowers” from “orange” to “red” generates the correct prediction. Background image: GQA dataset [4] (scene 31) under Creative Commons CC BY 4.0

**Incorrect label** – Instead of missing objects, relations, or attributes, the already provided information can also be incorrect. The scene graph then suggests that something other than the actual content is visible on the image, and answers related to these objects or connections are wrong. An example (Fig. 14) is that the “beard” of a man is incorrectly labeled as “bear.” Such scenes would require a correction of their scene graphs. The authors found this scene when using the scene browser with a filter for scenes containing the object “bear.” In this scene, they could not see a “bear.” Inspecting the ground truth scene graph showed that the object “bear” was located over the “beard” of a man. Such errors contribute to wrong predictions and can be corrected by selecting the scene graph node (using a right click) or the object on the right side of the tool and editing the provided information. Instead of incorrect node labels, attributes can also be wrong. Figure 15 shows an example where the authors asked for the color of the flowers. The flowers had the highest weight during prediction, and the model was sure that the flowers were orange. The label of the flowers also indicates that they are “orange;” however, a visual inspection of the image suggests that they are instead “red.” They corrected the attribute of the object “flowers” and received a correct prediction with 100% certainty. Similarly, they found examples where labels were not incorrect but not precise as well (e.g., “plant” vs “flower”) and, thus, needed correction. Incorrect labels for relations between two objects are additional sources for wrong predictions.

**Fig. 16:**
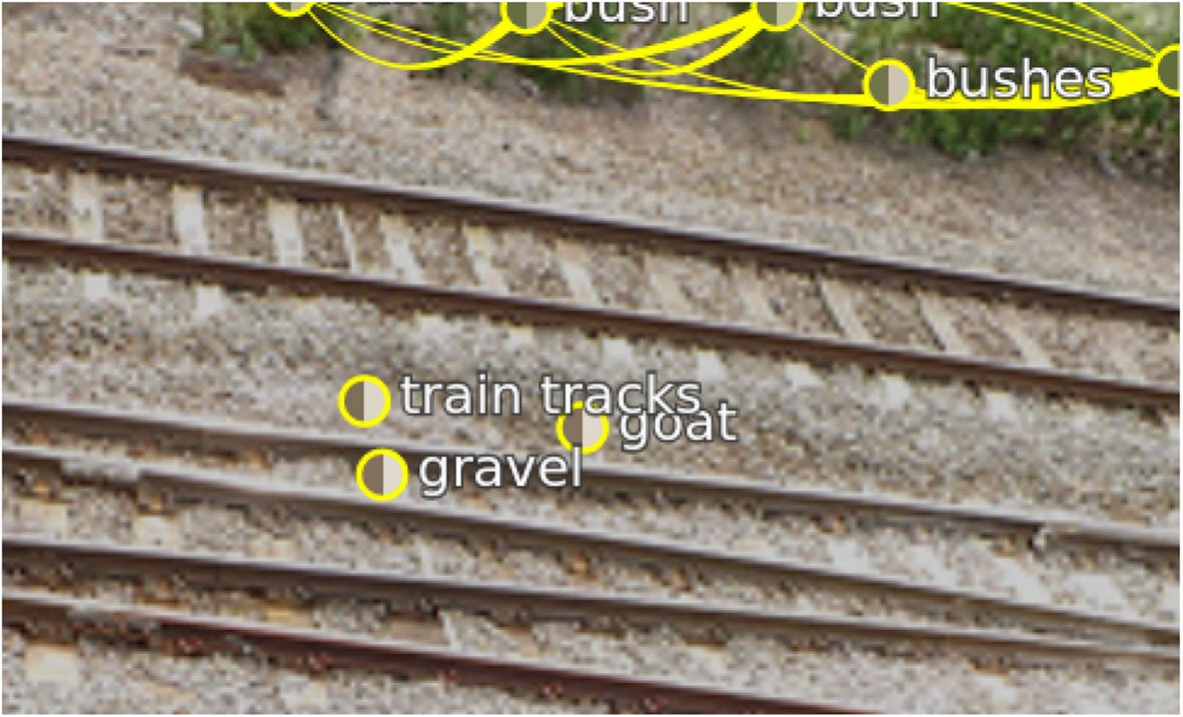
Erroneous data: Excerpt of a scene falsely containing the object “goat.” In the image, no “goat” is visible, but the scene graph contains an object labeled as “goat.” Background image: GQA dataset [4] (scenes 2369570) under Creative Commons CC BY 4.0

**Ghost object** – Sometimes scene graphs contain objects not available in the picture. An example is shown in Fig. 16 left. The ground truth question asked “Where is the goat?” and received an incorrect answer (visible with the evaluation browser). Verifying the scene graph showed that the node “goat” had no relation to other nodes. Additionally, the authors could not see the “goat” in the image. Sometimes, objects are tiny. However, no “goat” was visible even after hiding the scene graph. This seems to be a scene where both the ground truth scene graph contains a non-existent object, and the ground truth question asks for something unavailable in the image. They found other examples, where objects were available twice (but with different attributes) that also required correction.

**Fig. 17:**
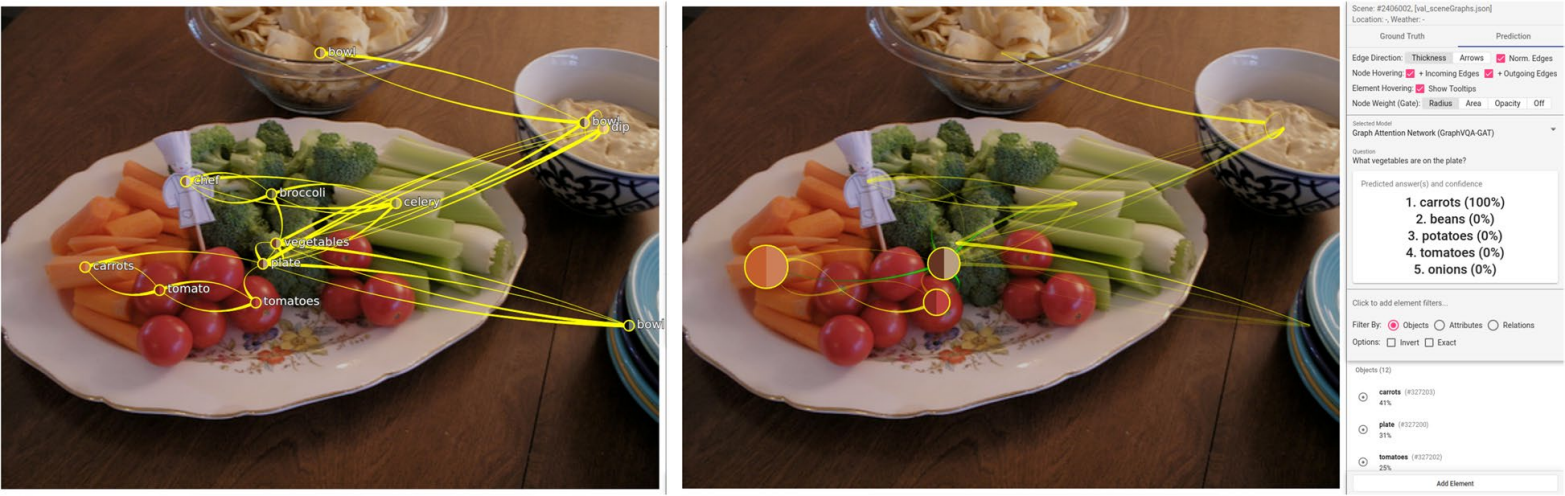
Unexpected prediction: The authors asked: “What vegetables are on the plate?” The model answers with “carrots” while the ground truth data expects “tomatoes.” Looking at the image shows that this answer is also correct. The ground truth scene graph visualization is on the left, and the prediction mode is on the right; it shows the edge attention convolution one. Background image: GQA dataset [4] (scene 2409347) under Creative Commons CC BY 4.0

**Fig. 18:**
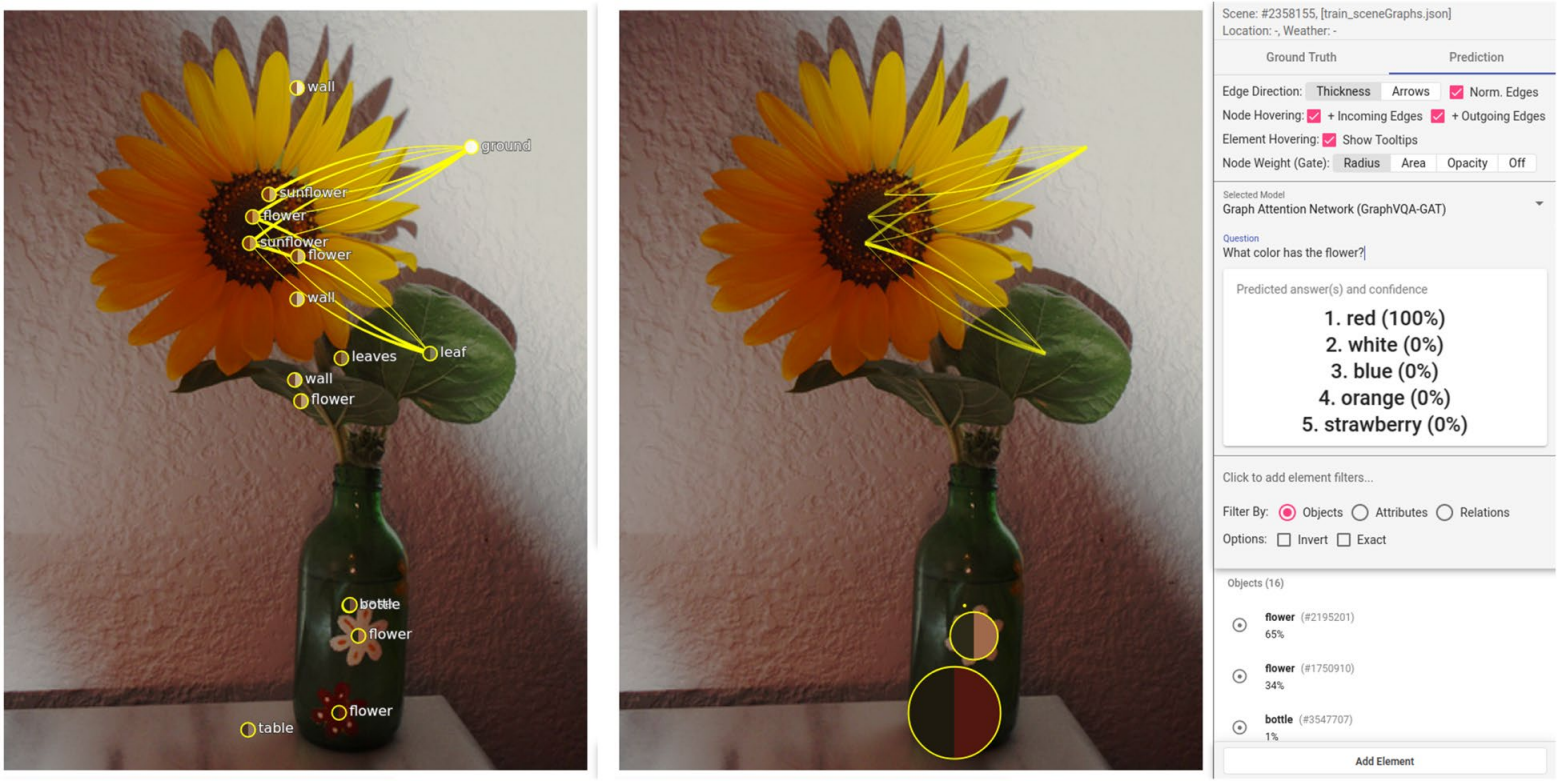
Unexpected prediction: Thinking of the large flower in the center of the image, the authors asked: “What color is the flower?” Unexpectedly, the model answered with “red.” Investigating the scene graph shows that a node at the bottom of the image is responsible for the answer. This node also represents a flower. However, it is smaller and drawn on the vase with the color red. The prediction view shows edge attention convolution one. Background image: GQA dataset [4] (scene 2358155) under Creative Commons CC BY 4.0

**Multiple correct answers** – A limitation of their model is that it can answer every question with only one term. Often, multiple different answers are possible. In such cases, the model selects one of the answers, and the others are listed as the following best predictions. In the evaluation browser, such predictions are shown as wrong because a different answer was expected. Inspecting such a case, a user quickly notices that the model also makes a correct prediction but not the one that was expected from the dataset based on the ground truth data. An example they found this way in the evaluation browser is shown in Fig. 17. The question asked was: “What vegetables are on the plate?” The expected ground truth answer from the dataset is “tomatoes” and the predicted answer “carrots” (100%). Inspecting the image shows that there are multiple vegetables on the plate, all of which would be correct answers. A similar but less expected behavior is shown in Fig. 18. The authors asked “What is the color of the flower?” Looking at the image, they expected “yellow.” Surprisingly, the model answered with “red.” The highest-rated node was also not the flower in the center of the image. There were small flowers that the authors did not immediately recognize, drawn on the surface of the vase. One of these flowers was “red.” In such a case, it would be good if the model had a way of prioritizing objects. Often, when posing a question, larger objects or those visible in the center are the subject of a question. However, using the scene graphs, the model cannot differentiate if an object is in the background, extremely small, or just partially visible in the image. Therefore, they cannot provide an acceptable solution to solve the problem to answer with the color of the flower in the center. Deleting the objects of the small flowers would lose information for other questions and would not be in their interest. Asking a more precise question, “What is the color of the sunflower?” generates the correct prediction (“yellow” (100%)).

#### Adding new scenes

The authors’ approach also supports adding new scenes to the dataset. Users are free to upload their images, create a scene graph, and pose their questions. In a next step, such scenes could be used to retrain the model. Basic features to add new scenes and their scene graphs are available in their system. Adding many new scenes would require additional features of their scene graph generation approach to speed up the process of annotation (e.g., automatic recommendations and copy functionality).

### Quantitative evaluation

The following evaluation results are briefly summarized from the authors’ previous work. A detailed description of the procedure and data acquisition is found in Schäfer et al. [9].

The authors base their analysis on token co-occurrence as an intuitive measure of node/object significance in the context of a question and a model prediction. They hypothesize that an input question will specify a (group of) target object(s) to which it refers, typically by name or by a distinguishing attribute. This leads to a statistically increasing token co-occurrence count with the input question. Similarly, if the model concludes an answer from a node in the graph, for example, a color or a material, the node statistically registers more token co-occurrences with the prediction than the nodes of lesser importance that the model did not base its answer on. In summary, nodes with higher token co-occurrence counts with input question or prediction are statistically important in the context of the question or the model’s generated answer. They then verify if the graph attention scores correlate with the importance of nodes in the respective context.

#### Summary of the results

The authors calculate the Spearman’s rank-order correlation coefficient ($r_s$) and the Pearson correlation coefficient ($r_{xy}$) for each result.

**Fig. 19:**
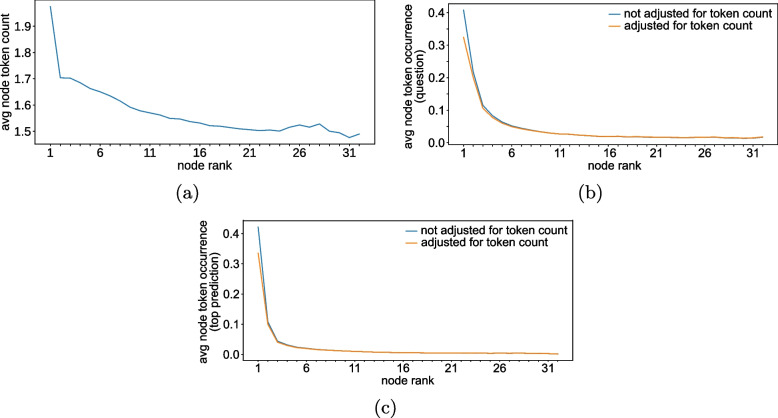
**a** Mean node token count *vs* node gate weight rank; **b** Mean co-occurrence ratio of node tokens with question tokens *vs* node gate weight rank; **c** Mean co-occurrence ratio of node tokens with top prediction token *vs* node gate weight rank

Figure 19a shows the mean token count of nodes for any given node rank. Among the top three ranks, a considerable decrease of 0.3 in the mean token count can be observed, $r_s(30) = -.94$ ($p < 0.001$); $r_{xy}(30) = -.80$ ($p < 0.001$).

The blue graph in Fig. 19b shows the mean co-occurrence count between node tokens and input questions for any given node rank, $r_s(30) = -.96$ ($p < 0.001$); $r_{xy}(30) = -.58$ ($p < 0.001$). Because higher token counts increase the probability of matches between tokens, the authors computed re-balanced scores based on the mean token count across all ranks. The orange graph in Fig. 19b presents the results, $r_s(30) = -.96$ ($p < 0.001$); $r_{xy}(30) = -.60$ ($p < 0.001$).

Table 1 shows in how many instances co-occurrences between node tokens and question tokens were recorded and in how many of these instances the node with the largest attention probability registered the highest number of co-occurrences. The overall value describes how frequently the highest ranked node is at least tied for the highest number of co-occurrences, regardless of whether any co-occurrences exist. Across the entire dataset, ties for the highest number of co-occurrences were recorded in $34.32\%$ of possible instances. In the reduced sample set, ties were slightly less frequent at $33.21\%$ of possible instances.

**Table 1:** In how many of the instances, co-occurrences between the input question and graph nodes were recorded, and in how many of these instances, the graph node with the highest gate weight (co-)registered the highest number of co-occurrences?

Question co-occurrence	Co-occurrence exist	Top rank : most co-occurrence
Full dataset	61.24%	54.59% (ovr: 33.44%)
Reduced set (size 6–32)	61.09%	54.74% (ovr: 33.44%)

Figure 19c shows the mean co-occurrence count between node tokens and top prediction for any given node rank, $r_s(30) = -1.0$ ($p < 0.001$); $r_{xy}(30) = -.45$ ($p = .010$). As with the previous figure, the authors computed re-balanced scores based on the mean token count across all ranks, $r_s(30) = -.99$ ($p < 0.001$); $r_{xy}(30) = -.47$ ($p = .007$). In both cases, the highest rank (i.e., highest node attention weight in the graph) correlates strongly with the number of token co-occurrences with the main prediction. The largest difference can be observed between the top three ranks. Compared to the co-occurrence with input question tokens (Fig. 19b), the immediate decrease is even more pronounced.

Table 2 shows in how many instances co-occurrences between node tokens and top prediction were recorded, and in how many of these instances the node with rank one registered the highest number of co-occurrences. The overall value describes how frequently the highest ranked node is tied for the highest number of co-occurrences, regardless of if any co-occurrences exist. Across the entire dataset, ties for the highest number of co-occurrences were recorded in $26.26\%$ of possible instances. For the reduced sample set, this value decreases slightly to $25.27\%$.

**Fig. 20:**
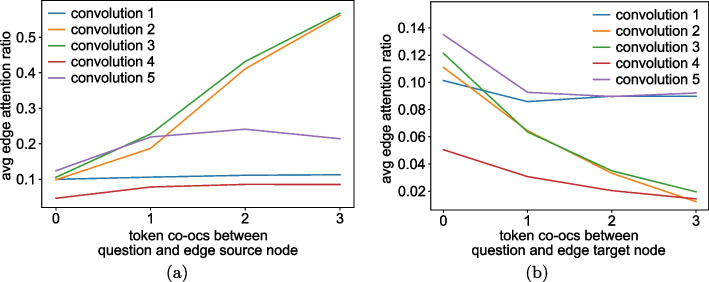
Mean edge attention ratio across all five GAT convolutions compared to the number of token co-occurrences between the (**a**) source node and (**b**) target node of the given edge and the input question

Figure 20a shows the mean edge attention ratio across all five GAT convolutions in relation to the number of token co-occurrences between the edge’s source node and the input question. Across all convolutions, few token co-occurrences appear to correlate with low edge attention. In the first convolution, edge attention does not appear to change in relation to co-occurrences at all. In convolutions two and three, edge attention appears to increase with the number of recorded co-occurrences. The last convolution exhibits a similar increase in edge attention for instances with zero to one co-occurrence while remaining stagnant around the higher co-occurrence counts. The fourth convolution features the lowest mean edge attention ratios overall and appears to be mostly stagnant. Only a small increase between zero and one token co-occurrences can be observed.

**Table 2:** In how many of the instances, co-occurrences between the top prediction and graph nodes were recorded, and in how many of these instances, the graph node with the highest gate weight (co-)registered the highest number of co-occurrences?

Prediction co-occurrence	Co-occurrence exist	Top rank : most co-occurrence
**Full dataset**	50.68%	82.26% (ovr: 41.69%)
w/o left/right	56.56%	82.26% (ovr: 46.53%)
w/o yes/no	78.46%	82.26% (ovr: 64.54%)
w/o yes/no/left/right	93.49%	82.26% (ovr: 76.91%)
**Reduced set** (size 6–32)	51.09%	82.47% (ovr: 42.13%)
w/o left/right	56.99%	82.47% (ovr: 47.00%)
w/o yes/no	78.65%	82.47% (ovr: 64.86%)
w/o yes/no/left/right	93.56%	82.47% (ovr: 77.15%)

Additionally, the authors recomputed co-occurrence frequencies while filtering out instances where the top prediction is either “yes,” “no,” “left,” or “right,” because these tokens never occur in the context of nodes

Figure 20b shows the mean edge attention ratio across all five GAT convolutions in relation to the number of token co-occurrences between the edge’s target node and the input question. The edge attention decreases as token co-occurrences increase across all five convolutions. As it was the case in the context of the source node, the fourth convolution registers the lowest edge attention ratios overall, with 2%–5% on average.

#### Discussion

The results indicate that the graph gate visualization is relevant for bridging question inputs with generated model predictions. Measured through token co-occurrence, the gate weight values appear to be strongly related to both model inputs and outputs, although the decline toward the lower ranks appears to be steeper for the outputs.

In approximately 74% of all cases, the highest number of co-occurrences between nodes and the top prediction is uncontested. Considering the high rate of co-occurrences given the opportunity (over 93%) and because the authors found a significant correlation between the number of co-occurrences with the top prediction and gate weight rank, where the highest ranking node will record the highest number of co-occurrences in most cases, the graph gate visualization serves as a useful visual indication of where to look first to trace the prediction.

Moving away from their earlier scenario, where a question describes the object that holds the desired answer, the dataset features many questions that only allude to the target object by describing another object and how the two are related to each other. Based on their results, the related object holding the answer to the question should generally receive a higher gate score from the model than the object that serves as an entry into the question-answering process.

Finally, the authors’ evaluation revealed that the edge attention score in the final graph convolution does not correlate significantly with the token co-occurrence.

### User study with experts

The authors performed a user study with experts to assess the usability and usefulness of their visual analysis approach. They invited five experts in visualization and five experts in NLP and DL who volunteered to participate in the study (no compensation). Participants were from the authors’ institution but not involved in the research for the paper except for participating in this user study.

**Fig. 21:**
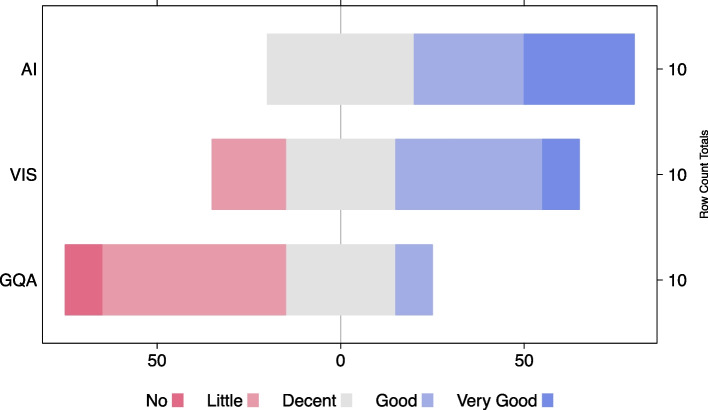
Self-assessment of the experts: Understanding of AI, VIS (Visualization), and GQA, measured using a five-step Likert scale

In the beginning of the study, each participant was asked to assess their knowledge of artificial intelligence, visualization, and graph-based VQA. The results of the self-assessment are presented in Fig. 21. Additionally, participants were asked if they were familiar with the GQA dataset to exclude bias. One participant answered yes, one participant did not answer, and all others answered no. All participants provided basic demographics, including age group and gender. Seven participants identified as male, and three identified as female. All participants were between 20 to 40 years old.

In the “Methods” section, the authors covered several use cases of how their tool can help identify and correct erroneous scenes graphs. Their tool includes two browsers, a scene browser and an evaluation browser, which can both be used to identify incomplete or faulty scenes. Once such a scene is found, the tool can be used to investigate, edit, and interact with the given scene. To aid the users in understanding a model’s internal workings, the authors visualize two internal states of the model using the scene graph in their model prediction view, i.e., edge scores, which depend on the GNN, and the global node attention pooling. In the “Discussion” section, the authors performed a quantitative evaluation to show that the internal states they chose to visualize correlate strongly with tokens occurring in the scene graph, question, and prediction.

The focus of this user study was to check the findings from the above-presented use cases and quantitative evaluation in a realistic application scenario. As VQA dataset, the training and evaluation split of the GQA dataset (see above) was used. Each expert was assigned two main tasks, performing typical dataset curation:

1. Use the scene browser and evaluation browser to identify erroneous scenes (R3) in the GQA dataset.
2. Correct a unique choice of three given erroneous scenes from a set of five using their visual scene graph editor, and interactive model embedding, including model state visualization (R3, R1).

#### Finding erroneous scenes

This task was divided into two parts: experts started using the scene browser to find two erroneous scenes in the GQA dataset, followed by using the evaluation browser with the same objective, or vice-versa; the order of the two parts was balanced in a random fashion to avoid any systematic bias. The decision to consider a scene as erroneous was left to the user, just as in an actual dataset curation project. However, the authors provided a set of guidelines that indicate issues in a scene graph, e.g., wrong or missing attributes, objects, and relations. They also instructed users that a scene graph did not have to include every object, relation, or attribute of the underlying visual ground truth. Coverage of the main scene elements, such as persons and objects in the foreground, and a background description were sufficient. The participants were instructed to keep this principle in mind when interacting with the VQA model as it could only answer based on the information included in the scene graph. They encouraged the participants to interact with the VQA model to identify problems in the scenes.

Before starting the task, the experts were briefed on the functionality of both the scene browser and the evaluation browser, including data sorting, filtering, and performance indicators.

**Fig. 22:**
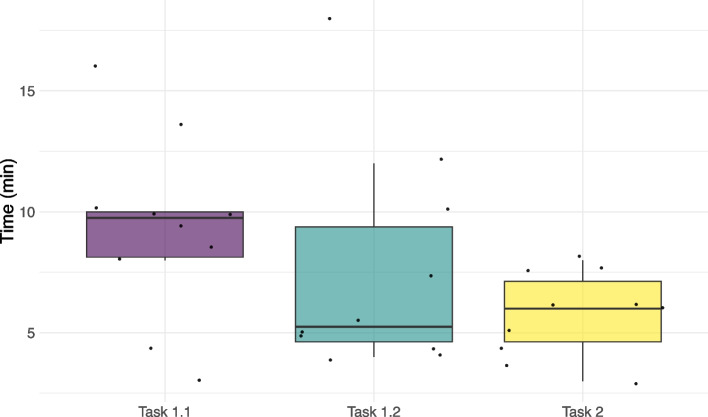
Task completion times for each task. Task 1.1: Use the scene browser to find two erroneous scenes. Task 1.2: Use the evaluation browser to find two erroneous scenes. Task 2: Correct three given erroneous scenes using the authors’ tool. Above measurements show that averaged over their sample group, completing Task 1 using the evaluation browser is faster than completing Task 1 using the scene browser

**Fig. 23:**
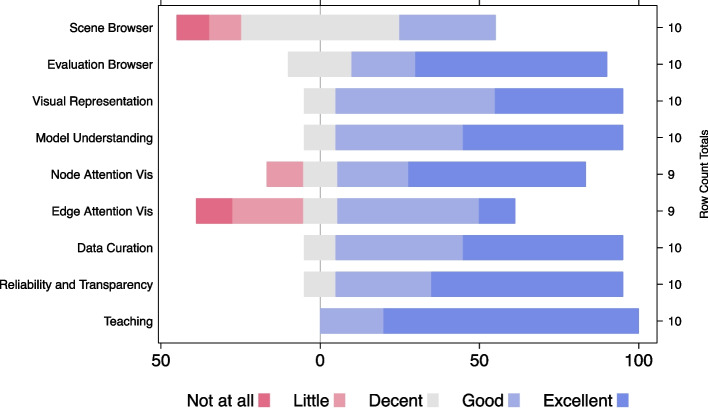
Aggregated expert opinion on different aspects of the authors’ visual analysis tool. From top to bottom: using the scene browser to find erroneous scenes; using the evaluation browser to find erroneous scenes; visual representation of the dataset and tool; the usefulness of the embedded model interaction with regards to dataset curation and model understanding; the usefulness of the node attention visualization regarding model and prediction understanding; the usefulness of the edge attention visualization regarding model and prediction understanding; applying the tool for dataset curation; using the tool to increase reliability and transparency of graph-based VQA; applicability of the tool for teaching purposes. Measured using a five-step Likert scale

Once an expert found two scenes considered erroneous, the time using the browser and the number of visited scenes were recorded. The results are presented in Fig. 22. Task 1.1 corresponds to the usage of the *scene browser* and Task 1.2 correspond to the usage of the evaluation browser. On average, the experts solved Task 1 in 9.5 minutes using the scene browser and 7.5 minutes using the evaluation browser. According to the subjective feedback, the experts preferred the evaluation browser to accomplish Task 1. This is shown in Fig. 23, rows one and two. Finally, the experts visited an average number of 2.9 scenes to accomplish Task 1 when using the scene browser, and an average of 2.2 scenes when using the evaluation browser.

#### Correcting erroneous scenes

In this task, experts were shown a random unique subset of size three out of a set of five hand-selected scenes to prevent a learning effect during the task. Each scene results in wrong model predictions, given a set of corresponding questions. Issues present in the scenes, leading to wrong predictions, include wrong attributes, wrong objects, and missing relations. The participants were then allowed to freely use the scene graph editor and embedded model to correct the scene. The goal was to generate a correct prediction for the given scene-question tuple. The indices of the five scenes and the associated questions used in this task are given in the supplemental material. Because the authors’ definition of erroneous and correct is based on the prediction result for a given question, an exact quantification of errors in the dataset is not necessary. Furthermore, an exact definition of a correct scene graph is often impossible, as scene graphs never contain all entities and relations. Therefore, the authors only consider correctness of a scene graph in the context of a question and the resulting prediction. However, even the correctness of a prediction is subject to interpretation by the user. To acknowledge this, they accept a small range of predictions as correct e.g. scooter or motorcycle as prediction for the question, e.g., “What type of vehicle?” In preparation for the task, the scene graph editing as well as all visualization features and the model interaction were explained to the experts using an example scene.

Similar to the previous task, the time to complete the task was measured. The results of Task 2 are presented in Fig. 22. Task 2 was solved in six minutes on average by the participants with little variation. One participant failed to correct a single scene because they decided to quit after five minutes; no further explanation was given. In the evaluation questionnaire, most experts considered the visual representation, specifically the frequent use of tooltips, the embedded model interaction, and node attention visualization as good or excellent. Only the edge attention visualization was considered to be of little or no use by a minority of experts. Results of the subjective feedback from the questionnaire are presented in Fig. 23.

#### Expert feedback

In addition to their measurements, the authors encouraged the experts to give feedback during and after the usage of their tool, implementing a think-aloud protocol [44]. A categorized summary of the feedback, excluding duplicates, is given below. The full original feedback is provided in the supplemental material.

Positive feedback:

- Hovering tooltips is excellent.
- Sorting and combined filter criteria are very helpful.
- The node attention visualization is very helpful.
- The evaluation browser is really good, the interface is easy to use and the node attention visualization is very useful.

Constructive feedback:

- Add a simplified view of the tool, eliminating the rarely used features.
- In large scenes, the amount of relations can be overwhelming. Selecting relations by group or type could help ease scene exploration.
- Some interactions could be polished.
- Color-coded relations and highlighting of impossible predictions would be helpful.
- Allow moving nodes around in the image plane for better alignment with the visual ground truth.

Critical feedback:

- The manual dataset curation task scales poorly on large datasets.
- Edge attention visualization is useless and hard to see owing to transparency.

The comments given by the experts are supplemented by the results of the authors’ evaluation questionnaire. Questions were answered on a five-option Likert scale ranging from not at all to excellent. The questions covered the usability, functionality, and visual representation of the tool. Additionally, the authors assessed the efficacy of the tool in dataset curation and teaching. The results of the questionnaire are presented in Fig. 23. A complete questionnaire with all questions is provided in the supplemental material.

#### Discussion of results

To find two erroneous scenes in the dataset as required in Task 1, the evaluation browser is suitable. This conclusion is based on multiple results and observations. First, the experts preferred the evaluation browser. Second, the experts completed the task faster using the evaluation browser. Third, the experts completed the task by visiting fewer scenes, equaling less false positives, using the evaluation browser. These results are elaborated on in the “Finding erroneous scenes” subsection.

In Task 2, the experts corrected faulty scenes using the authors’ scene graph editor and embedded model interaction and visualization. The authors conclude that their tool is suited for correcting faulty scenes in the VQA dataset. This conclusion is based on multiple results and observations. First, the experts considered the visual representation and visualization to be extremely helpful for solving Task 2. Second, the task completion time was short with little variation. Third, all scenes except one were successfully corrected. This result is consistent with the authors’ previous statistical analysis of the node attention and edge attention visualization that favored the node attention scores.

The participants can be partitioned in two groups based on their background: one half has a background in NLP and the other half has a background in visualization. Both groups were subjected to the same tasks, experiencing equal conditions and using the same dataset. In general, the authors did not observe any significant difference in experimental performance between the two groups. This includes task completion times, number of trials, and success rates. They attribute the similar performance of both groups to their extensive briefing on the subject and tool, including examples and extensive oral explanations on the area of research prior to the study. This allowed participants to start the study with an equal and high level of understanding.

In general, the experts considered the authors’ tool extremely useful for scene understanding, scene correction, and VQA transparency, specifically for teaching purposes. Apart from minor remarks about usability, the most relevant critique by the experts is the lack of scalability of manual dataset curation. This issue could be addressed in future work, for example, by integrating more automation into the curation process and considering the scalability of visualization approaches [45].

## Conclusions

The authors introduced a visual analysis approach for graph-based VQA models through an interactive and explorative tool (R3). Users can browse through a collection of scenes to identify scene graphs that can be filtered based on user preferences (R3). Once a scene is selected, the ground truth image and a visual representation of the scene graph are displayed (R1). The tool allows users to alter all crucial elements of scene graphs via adding, removing, and editing nodes, edges, and attributes (R2). The authors integrated models from GraphVQA [3] (R4) into their tool that were trained to perform graph-based VQA. However, the tool provides functionality to extend it to include other models as well (R5).

Their tool outputs the model predictions with a confidence score and visualizes two internal model states (R1). Because they deal with a graph classification problem, they enable their tool to visualize scores for each node that determine the importance of nodes.

The authors conducted an expert study, focused on dataset curation and model understanding using their visual analysis tool. The experts agreed that their tool was helpful to locate (R3) and correct (R2) erroneous scenes in the GQA dataset, the visual presentation was appealing, and the node attention visualization helped understand model behavior (R1). This supports the positive results of their quantitative analysis of the node attention visualization in the “Quantitative evaluation” section. Additionally, the experts considered their tool helpful to facilitate better understanding of VQA and well suited for teaching applications.

There are several ways in which this work could be improved and extended. Because the authors tested their approach in the context of data curation, the process of improving the dataset can be sped up in multiple ways. An automatic extraction of information from images could be used to spot mistakes and missing objects in the given scene graphs. A verification could compare if labels in scene graphs fit the content on the image and show identified problematic scenes to a user for final correction. Furthermore, automatically generated scene graphs could be used as a base for improvements by the user. Additionally, after finding faulty scenes, it might be helpful to identify similar scenes that might suffer from similar issues. The process of correcting scene graphs could also be improved. A better recommendation system and copy functionality could speed up the creation of large scene graphs. After correcting multiple scene graphs, a fine-tuning mechanism for the model could be implemented to improve its overall performance by re-training on the updated data samples. Additionally, in other graph convolution models similar information may be available in the final graph convolution. Such models could be investigated and used as a basis in the authors’ analysis system. Furthermore, a larger study with additional standardized questionnaires could be performed. This study could also provide stronger statistical evidence for their method.

## Supplementary information

Supplementary Material 1.

Supplementary Material 2.

## Data Availability

The authors’ approach is based on the source code of GraphVQA [3], available on GitHub at https://github.com/codexxxl/GraphVQA, and uses the GQA dataset at https://cs.stanford.edu/people/dorarad/gqa/; questions are available at https://downloads.cs.stanford.edu/nlp/data/gqa/questions1.2.zip, images are available at https://downloads.cs.stanford.edu/nlp/data/gqa/images.zip. The source code of the authors’ system is publicly available through DaRUS [10] at https://doi.org/10.18419/darus-3909 and on GitHub at https://github.com/Noeliel/GraphVQA-Explorer. The authors provide pretrained model parameters and evaluation data through DaRUS at https://doi.org/10.18419/darus-3597.

## Abbreviations

CV: Computer vision
DL: Deep learning
GAT: Graph attention network
GNN: Graph neural network
LSTM: Long short-term memory
ML: Machine learning
NLP: Natural language processing
NNs: Neural networks
Scene Graph QA: Scene graph question answering
VQA: Visual question answering
XAI: Explainable artificial intelligence

## Footnotes

1 https://github.com/Noeliel/GraphVQA-Explorer
