# Supplementary material for: Visual explainable artificial intelligence for graph-based visual question answering and scene graph curation
Source: Vis Comput Ind Biomed Art. 2025 Apr 7;8:9. doi: 10.1186/s42492-025-00185-y (PMC11977082; doi:10.1186/s42492-025-00185-y)
Supplement: Supplementary file 2 — Supplementary Material 2. [file 42492_2025_185_MOESM2_ESM.pdf]

Supplemental Material: “Visual Explainable AI for Improving Graph-Based VQA“

June 10, 2024

1 Supplemental Material for Section 4.3.3

1.1 Expert Feedback

We included slight corrections to spelling, grammar, sentence structure and technical terms in our expert feedback. In this supplemental, the revised expert feedback is compared to the raw, unedited feedback.

Positive feedback:

- Hovering tooltips are excellent. (“Hovering is an excellent design“)
- Sorting and combined filter criteria are very helpful. (“It is really helpful that you can sort ib the scenes / evaluation views“) (“The option to combine multiple filter criteria in evaluation view is nice to speed up searches“)
- The node attention visualization is very helpful. (“Viewing the entities that most influenced a decision was very helpful“)
- The evaluation browser is really good, the interface is easy to use and the node attention visualization is very useful. (“usability really nice“) (“interface was easy to use and appealing“)

Constructive feedback:

- Add a simplified view of the tool, eliminating the rarely used features. (”Maybe a simple view that doesn’t show all features could make the tool more approachable“)
- In large scenes, the amount of relations can be overwhelming. Selecting relations by group or type could help ease scene exploration. (“Sometimes, the amount of relations in a single scene looks a bit overwhelming . . . “) (“But maybe it could help to add a more visually interactive filtering aid, e.g. clicking on one type of relation removes all other types from the view“)
- Some interactions could be polished. (“. . . it is still in a prototype phase“) (“some interactions are less polished“)
- Color-coded relations and highlighting of impossible predictions would be helpful. (“color-code relations“) (“mark non-existing objects in answers“)
- Allow to move nodes around in the image plane for better alignment with the visual ground truth. (“ I would like to be able to move the existing node“)

Critical feedback:

- The manual dataset curation task scales poorly on large datasets. (Literal comment)
- Edge attention visualization is useless and hard to see due to transparency. (Literal comment) (“edgeweight vis not recognized“)

1.2 Evaluation Questionaire

In this supplemental are all questions asked in our evaluation questionaire. Each question has a single choice answer on a five element Likert scale (Not at all, Little, Decent, Good, Very Good).

1. Do you think the Scene Browser is helpful to find erroneous scenes?
2. Do you think the Evaluation Browser is helpful to find erroneous scenes?
3. Do you think the visual representation of the ground scene provides a clear and useful scene overview?
4. Do you think the embedded model interaction helps understanding model behav-iour and aids scene data curation?
5. Do you think the node weight visualization in the prediction view helps to under-stand model behavior and to find scene errors?
6. Do you think the edge weight visualization and animation in the prediction view helps to understand model behavior and to find scene errors?
7. Do you think our tool can be efficiently used to improve and curate scene graph da-tasets?
8. Do you think that this system could make the use of scene-graph-based VQA more reliable and transparent?
9. Could this system be beneficial for teaching purposes?

The final question allowed a text answer: What feedback can you give? (features, usability, design, etc.)

1.3 Scenes and Questions Used in Task 2 of the Study

The indices of scenes and associated questions used in task 2 are given listed below:

1. Scene, ID: 2416219, Question: On which side of the photo is the towel, the left or the right?
2. Scene, ID: 2333288, Question: What color is the sign?
3. Scene, ID: 2386906. Question: What color is the sink?
4. Scene, ID: 2369207, Question: Where is the onion?
5. Scene, ID: 146, Question: What type of vehicle?
